# A pyrosequencing insight into sprawling bacterial diversity and community dynamics in decaying deadwood logs of *Fagus* *sylvatica* and *Picea* *abies*

**DOI:** 10.1038/srep09456

**Published:** 2015-04-08

**Authors:** Björn Hoppe, Krüger Krger, Tiemo Kahl, Tobias Arnstadt, François Buscot, Jürgen Bauhus, Tesfaye Wubet

**Affiliations:** 1 Department of Soil Ecology, UFZ - Helmholtz Centre for Environmental Research, Halle (Saale), Germany; 2 Chair of Silviculture, Faculty of Environment and Natural Resources, University of Freiburg, Freiburg i. Brsg., Germany; 3 Department of Bio- and Environmental Sciences, TU Dresden, International Institute Zittau, Zittau, Germany; 4 The German Centre for Integrative Biodiversity Research (iDiv), University Leipzig, Leipzig, Germany

## Abstract

Deadwood is an important biodiversity hotspot in forest ecosystems. While saproxylic insects and wood-inhabiting fungi have been studied extensively, little is known about deadwood-inhabiting bacteria. The study we present is among the first to compare bacterial diversity and community structure of deadwood under field conditions. We therefore compared deadwood logs of two temperate forest tree species *Fagus* *sylvatica* and *Picea* *abies* using 16S rDNA pyrosequencing to identify changes in bacterial diversity and community structure at different stages of decay in forest plots under different management regimes. *Alphaproteobacteria, Acidobacteria* and *Actinobacteria* were the dominant taxonomic groups in both tree species. There were no differences in bacterial OTU richness between deadwood of *Fagus* *sylvatica* and *Picea* *abies*. Bacteria from the order *Rhizobiales* became more abundant during the intermediate and advanced stages of decay, accounting for up to 25% of the entire bacterial community in such logs. The most dominant OTU was taxonomically assigned to the genus *Methylovirgula*, which was recently described in a woodblock experiment of *Fagus* *sylvatica.* Besides tree species we were able to demonstrate that deadwood physico-chemical properties, in particular remaining mass, relative wood moisture, pH, and C/N ratio serve as drivers of community composition of deadwood-inhabiting bacteria.

Deadwood is an important structural component in forest ecosystems. It provides shelter and nutrition to various organisms, primarily fungi and saproxylic insects^1^,^2^,^3^. It also partakes in numerous ecosystem functions^4^, including carbon sequestration and nutrient cycling^5^,^6^,^7^,^8^. Many investigations have focused on the diversity and composition of fungal communities, their roles in wood decomposition^9^,^10^, and their interactions with different forest management regimes^11^,^12^. Conversely, the role of prokaryotes in deadwood and related ecosystem processes has only been examined in a few case studies such as the investigation into bacterial communities in deadwood of an East Asian pine species^13^ and the presence of coexisting bacteria with *Hypholoma fasciculare* sampled in seven tree stumps by Valášková *et al.*^14^. Substrate properties such as nutrient and water content have been shown to strongly influence wood colonization by microbes^10^,^15^. Greaves^16^ first developed a concept concerning a functional classification of wood-inhabiting bacteria: with (1) bacteria that affect permeability but do not cause losses in material strength, (2) bacteria that attack wood structures, (3) bacteria that act as integral synergistically members of the total microflora and (4) the “passive” bacteria, which may act as antagonists to other bacteria. Waterlogged and oxygen-depleted wood is mainly degraded by bacteria^17^ through a process that may be so slow that trees remain intact long enough to think of salvaging submerged logs^18^. However, they may be inefficient as wood decayers on their own, though bacteria, especially members of the *Actinobacteria* are expected to be among the early initial colonizers of deadwood and to permeate or even degrade its lignified cell walls via secretion of cellulases^19^,^20^,^21^. The composition of the primary wood-inhabiting bacterial communities may also be a consequence of the composition of the surrounding soil bacterial communities^22^. Associations among saprotrophic or wood-decaying Basidiomycota and bacteria in deadwood have been examined in several studies and reviews published over the last three decades^21^,^23^,^24^,^25^. Both antagonistic^25^,^26^ and mutualistic interactions^27^,^28^ have been observed during wood decomposition processes. A recent network analysis revealed non-random co-occurrence patterns of bacterial nitrogenase-encoding *nifH* genes with fungal sporocarps on deadwood logs of *Fagus sylvatica* and *Picea* *abies*^29^. This finding provided further evidence of potential mutualistic interactions between fungi and methylotrophic N-fixing bacteria that consume methanol, which is a by-product of enzymatic lignin degradation^27^,^30^,^31^. Hervé *et al.*^32^ investigated changes in bacterial community structure over time in deadwood inoculated with the lignin-degrading fungus *Phanerochaete* *chrysosporium*. They discovered variations in bacterial community composition across time to be incidental, but also identified members of the *Burkholderiaceae* to be always present in the mycosphere.

Apart from these studies, little is known about the community dynamics of wood-inhabiting bacteria during wood decay in temperate ecosystems. The study we here present is among the first to investigate bacterial diversity and community structure in deadwood under field conditions and applying deep 16S rDNA metabarcoding. Specifically, it compares the bacteria in deadwood logs of two common temperate timber tree species grown in geographic proximity, the deciduous *Fagus* *sylvatica* and the conifer *Picea* *abies*, at different stages of decay and under different forest management. Deciduous and coniferous woods have very different physico-chemical properties^33^,^34^. We anticipated that the structures of the bacterial communities would depend strongly on the properties of the deadwood and the identity of the tree species from which it derived. The primary objective of this study was therefore to determine which of these wood properties correspond or determine the composition and diversity of deadwood-inhabiting bacterial communities, and to identify the key players in the bacterial communities in the two deadwood types on at least the family level. In relation to a previously conducted study on the distribution of *nifH* genes in deadwood^29^, we additionally assumed that N-fixing bacteria from the order *Rhizobiales* were more abundant during the intermediate stages of wood decay, when fungal sporocarp richness is known to be highest and the provision of nitrogen is crucial^29^.

## Results

### Wood properties

In addition to assigning each log to a decay class, we determined their C and N contents, the concentrations of both elements per unit wood density (g/cm^3^), and also their relative wood moisture contents and pH values (Tables S2a and S2b). The C/N ratio in *Picea* logs ranged from 629.7 ± 48.4 to 422.9 ± 54.4 and was thus substantially greater and more variable than that in *Fagus* logs (376.9 ± 11.5 to 193.7 ± 15.6). In logs of both tree species, the C/N ratio decreased slightly with increasing decay class. This decrease was largely due to an increase in the logs' N concentration as they decayed (from 0.13 to 0.25% on *F. sylvatica* and 0.08 to 0.14% on *P.* *abies*, Table S2a). Due to the significant decrease of wood density (from 0.5 to 0.2g/cm^3^ on *F. sylvatica* and 0.35 to 0.15g/cm^3^ on *P.* *abies*), we also observed a decrease in total N and C content per density unit (Table S2a). There was no significant difference in wood moisture between the two tree species, but the moisture contents of both species' logs increased significantly with the extent of decay, from 52.2% ± 5.7 to 155.2% ± 9.1 in *F. sylvatica* logs and 48.7% ± 11.6 to 163.1% ± 24.6 in *P. abies*. Finally, the pH of *F.* *sylvatica* logs was significantly higher (*P* < 0.001) than that of *P. abies* logs; this trend was independent of the logs' state of decay.

### Sequence data analysis

In total, 125,183 reads were obtained from the 47 amplicon libraries by 454 pyrosequencing of the deadwood samples. Sequences were initially quality checked, trimmed (115,750 sequences), normalized per sample (1,837 reads per sample) and screened for potential chimeras. CD-HIT clustering of the remaining 73,099 sequences (discarding potential chimeras) yielded 7,388 OTUs at a 97% cutoff, 5,016 of which were singletons, 807 doubletons, and 368 tripletons. A total of 85 OTUs (among them 56 singletons) stemming from *Cyanobacteria* related to chloroplasts were also removed from the dataset.

In total, 61,831 sequence reads distributed to 1,180 OTUs were retained for analysis. Taxonomic assignments were achieved for 99.75% of these filtered OTUs (61,776 sequences (99.9%)) at the phylum level (including proteobacterial classes). 1,056 (58,498 sequences (94.6%)), 901 (53,732 sequences (86.9%)) and 633 (39,645 (64.1%)) OTUs were classified at the level of order, family, and genus level, respectively.

### Bacterial 16S rDNA diversity, richness and relationship with wood parameters

Species richness across all samples ranged from 105 to 378 OTUs with an average of 258 ± 56. We did not observe any significant differences in mean OTU richness between the two tree species (*P = * 0.52) (Fig. S2). One-way analyses of the variance in mean bacterial species richness for different decay classes revealed an increase from decay class 1 to 3 in *Fagus* logs (Fig. S2): the species richness in decay class 1 logs (191.6 ± 21.1) was significantly lower than in decay class 3 (291.9 ± 19.4). We did not detect any significant variation in OTU richness in *Picea* logs, although the species richness in the two later stages of decay (271 ± 17) was slightly higher than that in stages 1 and 2 (254 ± 14, Fig. S2). This increase is reflected in the observed correlations between OTU richness and the remaining mass of the logs (which provides a measure of their extent of decay). Bacterial OTU richness correlated significantly and positively with the extent of wood decay (*P* < 0.0005, *R^2^* = 0.45) in *Fagus* logs but not in *Picea* logs (*P* = 0.25, *R^2^* = 0.06) (Fig. S3).

**Figure 1:**
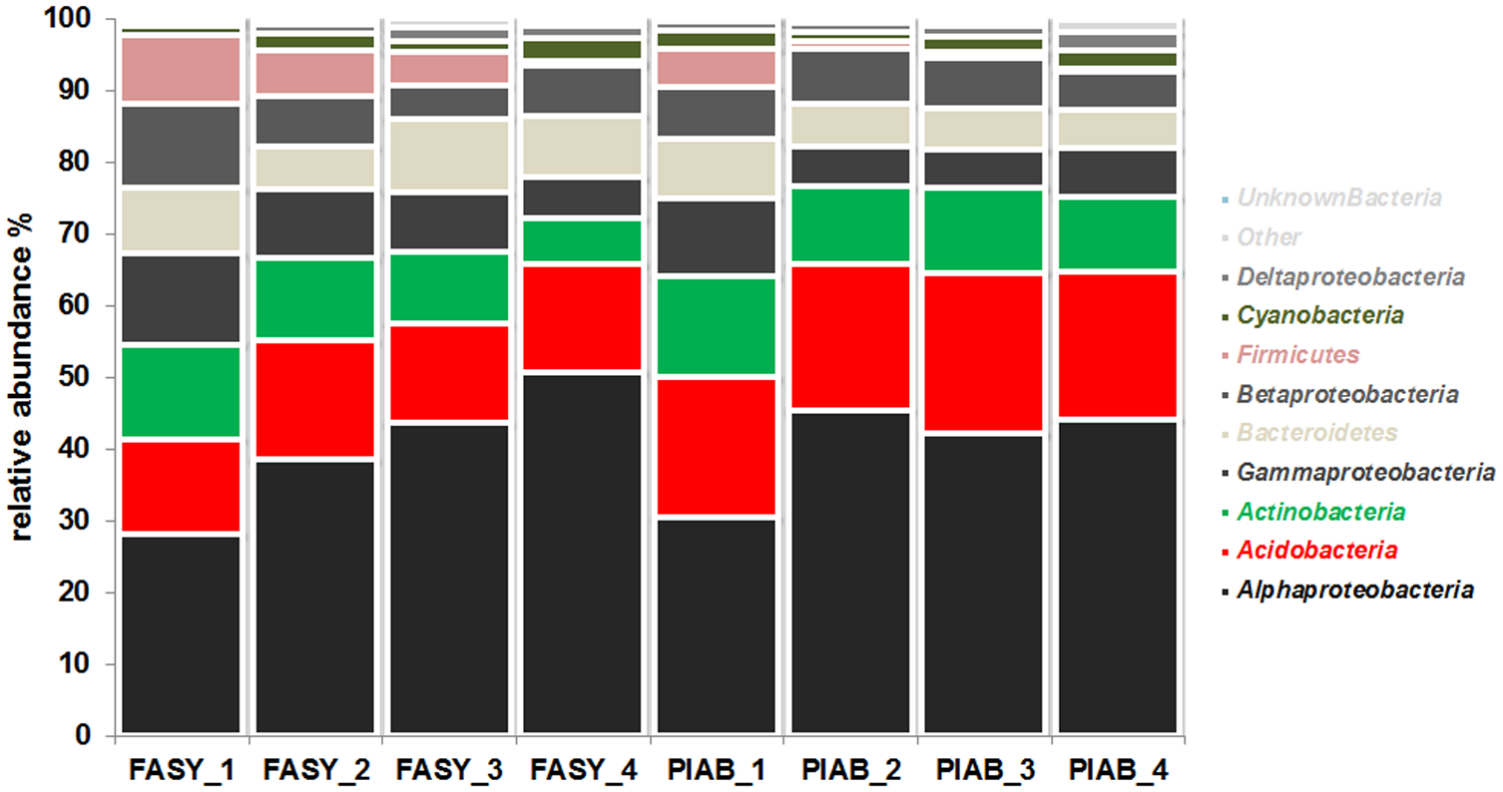
Relative abundances of phylogenetic groups (bacterial phyla including proteobacterial classes) in deadwood from two species (*Fagus* *sylvatica* = FASY, *Picea* *abies* = PIAB) in different stages of decay (decay classes 1–4). OTUs that could not be taxonomically assigned at the phylum/subphylum level are reported as “Others” and comprise ≤ 0.006% of all sequences. The category “other” also includes all OTUs with <1.5% relative sequence abundance.

The mean relative abundance of dominant bacterial phyla (including proteobacterial classes) did not differ greatly between the two tree species (Kronafiles SK1, SK2, SK3 in supplementary information). *Alphaproteobacteria* were dominant on both *Fagus* (38.9 ± 3.1%) and *Picea* logs (41.6 ± 2.1%); other abundant phyla included *Acidobacteria* (14.9 ± 2% and 20.9 ± 0.7%) and *Actinobacteria* (10.5 ± 0.7% and 11.4 ± 0.1%). However, there were significant differences in the relative abundances of these phyla between decay classes in logs of the same tree species (Fig. 1, Table S3). The relative contribution of *Alphaproteobacteria* increased from decay class 1 to 4 in both *Fagus* and *Picea* logs (from 28.1 ± 3% to 50.7 ± 1.9% and 30.4 ± 3.7% to 44.1 ± 1.5%, respectively) (Tab. S3). The relative abundances of *Acidobacteria* were consistent across decay classes but the contribution of *Firmicutes* decreased significantly from decay class 1 to 4 in both *Fagus* (9.6 ± 3.2% to 0.6 ± 0.2%) and *Picea* (5.2 ± 0.8% to 0.4 ± 0.2%) logs.

These shifts of relative abundances of the dominant phyla have further been observed performing rank abundance correlations (Table S4). This analysis revealed that the remaining mass per log had a significantly negative impact on the abundances of *Alphaproteobacteria* and *Deltaproteobacteria* (on *F. sylvatica* and *P. abies*), and *Cyanobacteria* (on *F. sylvatica*). In turn, *Actinobacteria*, *Firmicutes* (both on *F. sylvatica* and *P. abies*), *Gammaproteobacteria* (on logs of and *F. sylvatica*) and *Bacteroidetes* (on logs of *P. abies*) significantly decreased during wood decay. Analogous results were observed for the impact of relative wood moisture on the abundances of the respective phyla, since this parameter significantly increases with mass loss (see Table S2b). The abundances of *Alphaproteobacteria* (on *Fagus* *sylvatica*) and *Deltaproteobacteria* (on both tree species) correlated negatively and significantly to C/N in contrast to *Firmicutes*. Members of this phylum were also found to be positively and significantly impacted by higher pH on logs of *Picea* *abies*, whereas *Acidobacteria* correlated negatively and significantly to pH on logs of *Fagus* *sylvatica* (Table S4).

**Figure 2:**
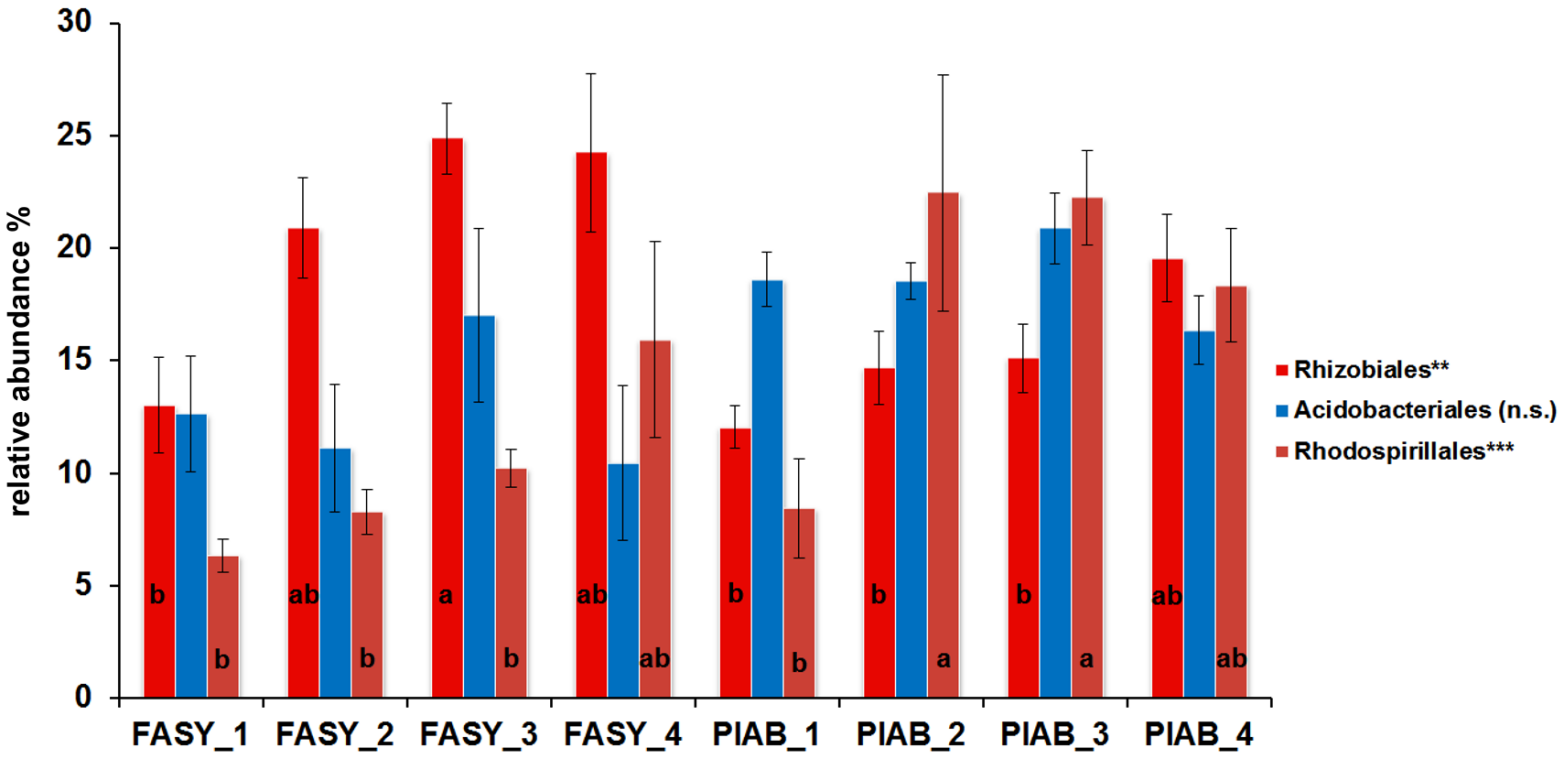
Relative abundances of the three dominant phylogenetic groups (bacterial orders) in deadwood of the two studied tree species (*Fagus* *sylvatica* = FASY, *Picea* *abies* = PIAB) in different stages of decay (decay classes 1–4). Differences between decay classes and tree species were analyzed by employing one-way analysis of variance and Fisher's Least Significant Difference (LSD) *post hoc* test (ns = not significant, * *P* < 0.05, ** *P* < 0.01, *** *P* < 0.001).

At the order level, members of the *Rhizobiales* were dominant in *Fagus* logs (22%), followed by *Acidobacteriales* (13%) and *Rhodospirillales* (11%) (Kronafiles SK1 and SK3 in supplementary information, Fig. 2). In *Picea* logs, *Rhodospirillales* (20%) were dominant, followed by *Acidobacteriales* (18%) and *Rhizobiales* (17%). As shown in Fig. 2, we also observed a significant increase of *Rhizobiales* from decay class one to three in *Fagus* logs (15.4 ± 5.8% to 26.9 ± 10.1%). Similarly, for *Picea* deadwood, the contribution of *Rhizobiales* went from 14.4 ± 6.4% in decay class 1 to 19.9 ± 6.6% in decay class 4. The most abundant OTU in our dataset appeared to be affiliated with the methanotrophic *Methylovirgula* genus from the family *Beijerinckiaceae*. In addition, there were another 5 highly abundant OTUs that were assigned to methanotrophic bacteria of the genera *Methyloferula, Methylocella* and *Methylocystis*. Pearson rank correlations revealed that *Methylovirgula* abundance had a significant negative correlation with the C/N ratio in *F.* *sylvatica* logs (*R^2^* = 0.38, *P* = 0.04) (Table S5). We also found that the abundance of *Methylovirgula* and *Methyloferula* correlated significantly and negatively with the remaining log mass but positively with the logs' moisture content (Table S5). No such correlations were observed for *Picea* logs. Fig. S4 further clearly illustrates the increase in the relative abundance of this dominant OTU as decay proceeds.

### Effect of forest management on bacterial richness

Our results also revealed a significant impact of forest management regimes on bacterial OTU richness. The analysis of forest management regimes' influence was based on separated data for *Fagus* and *Picea* logs in the respective forest plots. When considering *Fagus* logs in spruce plots, and *vice versa* (Fig. S5), we observed that the highest OTU richness in *Fagus* logs was in the unmanaged plots (*P* < 0.001). While the *Picea* logs with the greatest OTU richness were those in managed spruce forests (269.2 ± 14.5), their richness was not significantly different to that for spruce deadwood in managed beech forests (248.2 ± 17.1) or unmanaged beech forests (266.8 ± 12.1). We also found that the richness of bacterial OTUs in *Fagus* logs was negatively associated with land use intensity (Fig. S6) by calculating its correlations with the intensity indices SMI (*R^2^* = 0.31, *P* = 0.02) and ForMI (*R^2^* = 0.21, *P* = *0.07*).

### Bacterial community structure and variation with progressing wood decay

**Figure 3:**
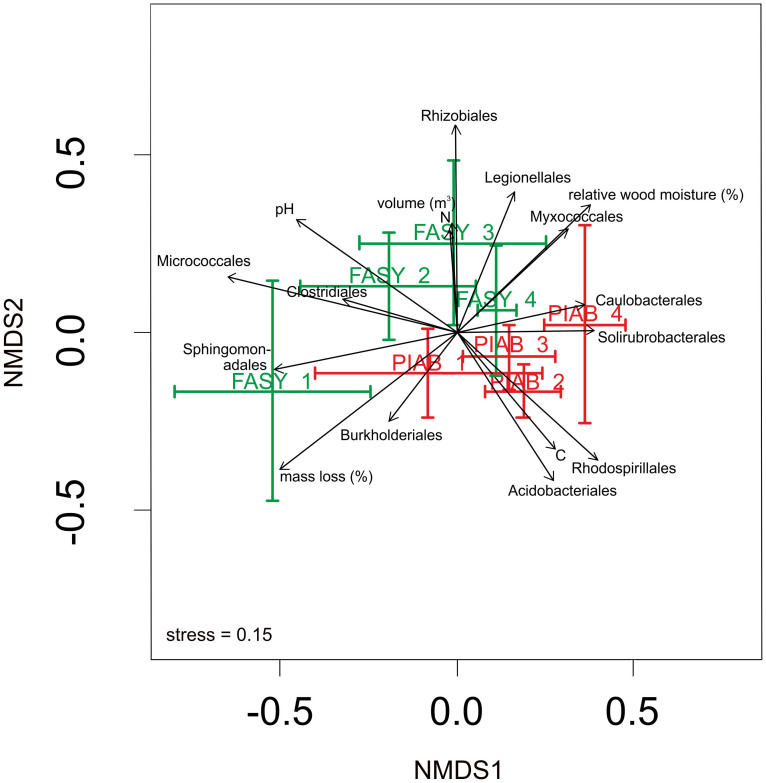
Two-dimensional non-metric multidimensional scaling (NMDS) ordination plots of bacterial community structure across the different tree species at each stage of decay (FASY1-4, PIAB1–4). Plots show centroids within a single decay stage, bars represent one SD along both NMDS axes. Statistical significances (*R^2^* and *P*-values) are based on Goodness-of-fit statistics for environmental variables and bacterial order abundances per sample.

As revealed by perMANOVA and NMDS tree species and decay classes significantly explained the observed variation in bacterial community structure (*P* = 0.0001), while forest management type did not (Table 1, Fig. 3). The importance of tree species was further confirmed with one-way ANOSIM using either relative abundance (Bray-Curtis or Euclidean) or presence/absence (Jaccard or Sörensen) data (Table S6; always *P* = 0.001). Furthermore, physico-chemical properties of the different types of deadwood were found to correlate significantly with bacterial community structure (as presented by dominant bacterial order; Fig. 3). Specifically, the decay class, remaining mass, volume, density, relative wood moisture, pH, C/N ratio and the concentrations and contents of C and N in the logs contributed significantly (*P* = 0.043-0.0001) to the observed variation in bacterial community structure in both tree species (Table 2). At the tree species level, bacterial community structure correlated significantly with decay class, remaining mass, wood density, C/N ratio and C content*.* Wood volume, C concentrations, N and C content and pH were important in *Fagus* deadwood, while the C/N ratio contributed significantly to explaining the variation in bacterial community structure in *Picea* deadwood (Table 2).

Similarity percentage analysis (SIMPER) for bacterial families revealed contrasting patterns for the two tree species/decay classes. In *Fagus* logs, members of *Acidobacteriaceae* (*Acidobacteria*) explained roughly 17% of the community variation among the 4 decay classes (Fig. S7). *Burkholderiaceae* (*Betaproteobacteria*), which accounted for 5% of the total bacteria on *Fagus* logs*,* explained roughly 9% of the total variation across different decay classes. This family appeared to be dominant in decay class 1 logs. The opposite pattern was observed for the family *Beijerinckiaceae,* which explained 8.5% of the community variation and which was almost absent in the earliest stage of decay but became much more abundant as decay progressed (Fig. S7). This family is represented by the most abundant OTU, which was assigned to *Methylovirgula*. In *Picea* logs, members of the *Acetobacteraceae* (*Alphaproteobacteria*) explained 20% of the total community variation across all decay classes. They were most dominant in decay class 2 logs. *Acidobacteriaceae* were consistently present at all decay stages and explained 10% of the community variation. They were the dominant bacterial family at all decay stages. *Burkholderiaceae* and *Beijerinckiaceae* explained 7 and 6.6% of the community variation, respectively, but there were no clear trends in their abundance comparable to those observed for *Fagus* logs.

## Discussion

We here present one of the first field studies on 16S rDNA bacterial community structure in deadwood of two Central European tree species using 454 pyrosequencing that provides an extensive insight into the diversity and composition of deadwood dwelling microorganisms on a rather underexplored substrate. Using a very comprehensive dataset of physico-chemical wood properties, we were able to identify key wood properties that correlate with bacterial community structure and abundances of dominant bacterial phyla (including proteobacterial classes). In line with the results of our recent study on the distribution of *nifH* genes^29^, we found that members of the order *Rhizobiales* became significantly more abundant during the intermediate to advanced stages of decay, indicating that they may play an important ecological role and contribute significantly to N-cycling.

We did not observe any difference in bacterial richness between the two tree species but did find that richness potentially increased as decay progressed (on logs of *Fagus* *sylvatica*). It is difficult to put these findings into context due to the absence of comparable data sets. However, the steady increase in bacterial richness as decay proceeds is consistent with results on fungal species richness obtained using the molecular techniques employed in this work^9^,^35^.

Our study also revealed a significant impact of forest management on bacterial OTU richness, which was significantly higher in *Fagus* logs in the unmanaged beech forest plots than in *Fagus* and *Picea* deadwood in the respective managed plots. This suggests that the bacterial communities in unmanaged forests are more species-rich, which may be due to a higher level of substrate continuity arising from the absence of wood extraction^12^. When comparing *Fagus* logs in managed beech stands to their counterparts in managed spruce stands (and *vice* *versa* for *Picea* logs), we also discovered a significant impact of the surrounding stand structures on the bacterial richness. Nacke *et al.*^36^, who studied soil 16S rDNA diversity in grassland and forest plots using the same experimental platform also detected a significantly higher Shannon diversity index in unmanaged beech plots than managed beech and managed spruce plots. In contrast to Purahong *et al.*^12^ who observed a higher fungal diversity in *Fagus* logs in managed beech stands than in *Picea* logs in managed spruce stands, we did not find such a clear pattern for bacterial OTU richness.

*Alphaproteobacteria*, followed by *Acidobacteria* and *Actinobacteria* were the dominant phyla in this study. The relative abundances of these phyla are similar to those observed by Nacke *et al.*^36^ in forest soils and in a separate study on decayed wood samples^14^. The relative abundance of *Acidobacteria* in *Picea* logs (20.9%) was higher than that in *Fagus* (14.9%). A previous study using a clone library sequencing approach^13^ similarly revealed members of the *Proteobacteria* to be dominant on *Keteleeria* *evelyniana* deadwood, followed by *Actinobacteria* and *Acidobacteria*. In contrast, *Bacteroidetes* was the second most abundant phylum (rather than *Acidobacteria* or *Actinobacteria*) across a range of samples studied in a different set of wood colonization experiments^22^. The most abundant OTU in this study was assigned to *Mucilaginibacter* (*Bacteroidetes*), a genus that has been shown to contain degraders of pectin and xylan^37^. This genus was also represented by the 13^th^ most abundant OTU in our dataset. Its abundance in *Fagus* logs increased continuously from 14.6% to 26.7% from decay classes 1 to 4 but in *Picea* logs it decreased from 25.3% to 15.2% with increasing decay stage. In contrast to the findings of Hervé *et al.*^32^, the genus *Dyella* of the *Xanthomonadaceae* only contributed marginally (0.02%) to the total sequence dataset.

Although *Actinobacteria* were expected to be among the dominant important early colonizers of deadwood^21^, they contributed only 10.5% and 11.4% of the total bacterial richness in logs of *Fagus* and *Picea*, respectively. However, we found that their relative abundance decreased significantly with progressing wood decay confirming their potential role in the early colonization and decomposition of dead wood logs. We assume that their dominant detection in culture-based studies may arise because of culturing conditions favoring them and because of fast germination of the dormant spores under conducive culturing conditions.

## 

In contrast to soils, where pH might serve as predictor for bacterial community structure and as a determinant for relative abundances of dominant bacterial phyla^38^,^39^ we did not observe such an important impact of pH in deadwood. In fact, we found negative correlations for *Acidobacteria* (on *P. abies*) and *Alphaproteobacteria* (on *F. sylvatica*), which have also been reported for soils^36^,^39^, but the rather small variance of pH between different wood species and its independency from decay classes does not substantially explain the shifts of the relative abundances of the dominant phyla. Our results rather reveal that the remaining mass or the respective decay class could be used as a potential predictor for the shifts in bacterial abundances in deadwood. However, since they are determined by significant variations of the wood physico-chemical properties (e.g. C and N concentration, relative wood moisture, density; compare Tables S2a and S2b), we assume that variations in the abundances of phyla are rather determined by a combination of wood properties than by single parameters, such as pH, alone.

In line with our assumptions, *Rhizobiales* was the dominant order in the intermediate and advanced decay classes. In fact, they accounted for almost 25% of the total sequence abundance in *F. sylvatica* logs of decay class 3. The relative contribution of *Rhizobiales* in the wood block experiments of Folman *et al.*^25^ and de Boer and van der Wal^24^ was also around 25% even though the total number of wood-inhabiting bacteria in that study was reduced by the bactericidal effects of the white-rot fungus *Hypholoma* *fasciculare*. The high abundance of *Rhizobiales* in that case was attributed to a mutualistic/predatory interaction since they were not detected on wood blocks without the fungus. The hypothesis that wood-degrading fungi meet their N requirements by associating with N-fixing bacteria was first raised by Cowling and Merrill^40^. This hypothesis has evolved over time, and microbial N-fixation in deadwood has since been demonstrated in several studies conducted over the last few decades^41^,^42^,^43^,^44^. This hypothesis was further corroborated by the detection of methanotrophic bacteria in deadwood that utilize methane as their sole carbon source^45^ and possess the *nifH* gene encoding the enzyme dinitrogenase reductase, which is important for dinitrogen fixation in some taxa^31^,^46^. Lenhart *et al.*^47^ identified eight saprotrophic fungi that produce significant amounts of methane under oxic conditions in the absence of methanogenic archaea, further supporting the possibility of mutualistic interactions between fungi and methanotrophic N-fixers.

Interestingly, the most abundant OTU in our dataset was taxonomically affiliated to the genus *Methylovirgula*, whose type strains Vorobev *et al.*^31^ isolated from the beech woodblocks studied by Folman *et al.*^25^. These methylotrophic bacteria are specialized to utilize methanol as their sole carbon source. Partial sequences of the *nifH* gene from these strains provided evidence of their N-fixing potential. We found that the abundance of *Methylovirgula* in *Fagus* logs correlated significantly and negatively with the C/N ratio (Table S5). Together with the preferential occurrence of this genus during the advanced stages of decay (Fig. S4), this finding supports the hypothesis that wood-decaying fungi interact mutualistically with certain bacterial taxa while suppressing or disfavoring others^21^,^24^. This is further supported by the results of Brunner and Kimmins^42^, who studied deadwood from two coniferous tree species in various stages of decay and found that nitrogenase activity was highest in decay classes 3 and 4. Their data indicated that the total rate of N fixation in such logs was up to 2.1kg N*ha^−1^*a^−1^.

Tree species was found as the main factor shaping the bacterial community structure in deadwood. This finding is comparable with the results of previous studies on bacterial community structure in forest soils beneath different tree species^36^,^48^. We also found that decay class significantly contributed to the shift in the bacterial community structure unlike the forest management regime. This is entirely consistent with our previous observations on the distribution of *nifH* genes within the bacterial communities in the same logs^29^. The observed effect of the tree species mainly attributed to the wood physico-chemical properties (Table 2 and the corresponding attributes in Table S2a). Among these parameters we were able to identify pH, C and N availability, and wood moisture as the main determinants corresponding to drivers of the bacterial community structure in deadwood. These findings can also be compared to the results of recent studies that verified the impact of soil chemical properties (especially pH) on the corresponding bacterial^36^,^39^,^48^ and fungal^49^ communities.

This paper describes the bacterial diversity and community structure in deadwood of two tree species. The results presented in this study elucidate the functional traits of specific bacterial taxa involved in wood degradation. We have demonstrated that the deadwood bacterial community structures are influenced mainly by the trees species and specifically by wood's physico-chemical properties, where the most important drivers were remaining mass, density, pH, water content and C/N ratio. We also found that members of the order *Rhizobiales* were dominant in the studied deadwood logs, accounting for up to 25% of all bacteria present during the intermediate and late stages of decay. The most abundant OTU in our data set was assigned to the genus *Methylovirgula*, which has been shown to contain the *nifH* gene. This further supports the hypothesis that microbial N-fixation plays an important role in wood decomposition.

## Methods

### Experimental design, deadwood selection and physico-chemical properties

The study was conducted on plots of the German Biodiversity Exploratories^50^ located in the “Schwäbische Alb” UNESCO Biosphere Reserve in southwestern Germany according to the sampling scheme displayed in Hoppe *et al.*^29^. We surveyed deadwood logs in nine very intensively investigated 1ha plots (VIPs), with three plots representing three different forest types and management regimes, respectively: (i) unmanaged beech forests, where timber harvesting stopped several decades (20–70 years) ago, (ii) managed beech forests dominated by *Fagus* *sylvatica* and (iii) managed spruce forests dominated by *Picea* *abies,* which in both cases are characterised by uniform tree species composition, forest structure and site conditions. In April 2009, a set of 48 logs, equally representing the two tree species (24 logs per tree species (*P.* *abies* and *F.* *sylvatica*) located on the forest floor were randomly selected and their properties (length, diameter, tree species, e.g.) were characterized (see Table S1). The logs were selected to ensure that some of the *Fagus* logs were located in *Picea*-dominated plots and *vice versa*. In June 2009, 3–7 wood samples were collected from each log (according to log length; compare Purahong *et al.*^12^ and supplementary information) using a cordless Makita BDF451 drill (Makita, Anja, Japan) equipped with a 2 × 42cm wood auger as described by Hoppe *et al.*^29^ (further details provided in supplementary information; Fig S1). The upper surface layer and bark of the deadwood was removed to avoid external bacterial contamination. Prior to analysis, the wood samples were weighed, dried at 60°C to constant mass, and reweighed. The concentrations of C and N in wood samples were determined by total combustion using a Truspec elemental analyzer (Leco, St. Joseph, MI, USA). The samples' densities and relative wood moisture contents were calculated based on their dry masses. Sample pH values were determined by shaking 1g of dried wood in 10mL of distilled water for 120 minutes and measuring the pH of the resulting aqueous extract. Each deadwood log was assigned to one of 4 decay classes based on its remaining mass (%) by k-means cluster analysis as described by Kahl *et al.*^5^ and Hoppe *et al.*^29^. Higher decay classes corresponded to more extensive decay (Tables S2a and S7).

### DNA isolation, PCR and pyrosequencing

Total community DNA was isolated from 1g of each previously homogenized wood sample using a modified CTAB-protocol^51^ as described by Hoppe *et al.*^29^. All DNA extracts from the wood subsamples of the 48 logs were pooled into a composite extract prior to PCR amplification. In total, 47 16S rDNA gene amplicon libraries were obtained while no amplified product was found from one sample of *Fagus* *sylvatica*. For the amplicon library production we used fusion primers designed with pyrosequencing primer B, a barcode and the primer 341f^52^ as a forward primer and pyrosequencing primer A and the 907r^53^ to amplify the V3-V5 region of the eubacterial 16S rDNA gene. The primers were barcoded with a set of 10nt MID-barcodes provided by Roche (Roche Applied Science, Mannheim, Germany). For each composite DNA extract the amplicon libraries was amplified separately by PCR in triplicate 50µl reaction mixtures containing 25 µl 2x GoTaq Green Mastermix (Promega, Madison, WI, USA), 25 µM of each primer and approximately 20 ng template DNA. PCR was performed with an initial denaturation period of 1 min at 98°C followed by 30 cycles of 95°C for 45 s, 57°C for 45 s, and 72°C for 1 min 30s, then a final elongation step at 72°C for 10min. After checking the quality of the PCR products by separation on a 1.5% agarose gel, the replicates were pooled and purified by gel extraction using the QIAquick Gel Extraction Kit (QIAGEN, Hilden, Germany). The purified DNA was quantified using a fluorescence spectrophotometer (Cary Eclipse, Agilent Technologies, Waldbronn, Germany). An equimolar mixture of each library was subjected to unidirectional pyrosequencing from the 907r ends of the amplicons, using a 454 Titanium amplicon sequencing kit and a Genome Sequencer FLX 454 System (454 Life Sciences/Roche Applied Biosystems, Mannheim, Germany) at the department of Soil Ecology, UFZ.

### Bioinformatic Analysis

We performed multiple levels of sequence quality filtering. The 454 bacterial 16S sequences were extracted based on 100% barcode similarity. Sequences were trimmed from barcodes and sized to a minimum length of 350nt to cover the V4-V5 region of the 16S rRNA gene using mothur^54^. Sequence reads with an average quality score of <20, bases and homo-polymers of >8 bases were removed. Unique good quality sequences from the dataset were filtered and checked for chimeras using the uchime algorithm, as implemented in mothur. To avoid sampling size effects, the number of reads per sample was normalized to 1,837 for each data set by randomly subsampling to the lowest number of reads among samples.

The revised complete sequence dataset was then clustered and assigned to OTUs using CD-HIT-EST of CD-HIT version -4.5.4^55^ at a 97% threshold of pairwise sequence similarity. We used GAST (global alignment for sequence taxonomy)^56^ to taxonomically assign the OTUs against the arb-silva database 114^57^ as downloaded in August 2013.

### Statistical analysis

We performed a procrustes analysis^58^ using the *protest* function in vegan^59^ to test the impact of excluding the rare taxa (singletons, doubletons, tripletons). Procrustes analysis indicated that removing of rare taxa had no effect on the results obtained (procrustes correlation *R* = 0.94, *P* = 0.001), so singletons, doubletons, and tripletons were discarded prior to further analysis. OTUs stemming from *Cyanobacteria* related to chloroplasts were also discarded (85 OTUs incl. 56 singletons).

The effects of tree species, decay class and forest management type on the 16S OTU community structure in the sampled logs were analyzed by perMANOVA. ANOVA was used to assess the influence of tree species and decay class on bacterial OTU richness and the two richness estimators. We performed one-way analysis of variance (ANOVA) to identify significant (P < 0.05) differences between mean OTU richness in association with the respective tree species. ANOVA was coupled with Shapiro-Wilk's W test for normality and Levene's test for equality of group variances. In addition, we assessed the impact of forest management intensity on bacterial diversity using two land use intensity indices, SMI (Silvicultural Management Intensity indicator)^60^ and ForMi (Forest Management Intensity index)^61^, that were developed in parallel within the Biodiversity Exploratories research community. Pearson Rank correlations between the relative abundances of dominant bacterial phyla (including proteobacterial subphyla) and selected wood properties were performed in PAST^63^. One-way analysis of similarities (ANOSIM) calculations based on four commonly used distance measures in conjunction with data on OTU abundance and presence/absence was performed in PAST^63^ to test for significant differences in bacterial community structures and compositions among different tree species and decay classes, respectively. Assessments of statistical significance were based on 999 permutations and *P* values were Bonferroni-corrected. NMDS was conducted in R to investigate the influence of the following wood physico-chemical parameters on bacterial community structure: decay classes, the concentrations of the macronutrients C and N, relative wood moisture, wood density, remaining mass, and pH. Goodness-of-fit statistics (R^2^) for environmental variables fitted to the NMDS ordinations of the bacterial communities were calculated using the *envfit* function of the “vegan” package, with *P* values being based on 999 permutations^60^. Similarity Percentage analysis (SIMPER) was performed in PAST^62^ to identify the important bacterial families responsible for the observed clustering of samples.

## Author Contributions

B.H., D.K., T.K., J.B. and F.B. conceived and designed the study; B.H., T.A. and T.K. performed the field and laboratory work; B.H. and T.W. analyzed the data; B.H. and T.W. wrote the paper. All authors reviewed and edited the manuscript and also provided input for the discussion.

## Additional information

Data accessibility: The raw sequence data are available from the NCBI Sequence Read Archive (http://www.ncbi.nlm.nih.gov/Traces/study/) under experiment SRX589509. Corresponding MIDs and metadata are provided in Table S7.

## Supplementary Material

Supplementary Information Supplementary Information

## Figures and Tables

**Table 1:** Results of perMANOVA analysis of the Bray-Curtis dissimilarities for bacterial OTU community structure in relation to tree species, decay class (assigned based on the remaining mass of the log in question), management regime, and their interaction, Df = degrees of freedom; SS = sum of squares; MS = mean sum of squares; Pseudo-F = F value by permutation. Bold face indicates statistical significance (*P* < 0.05); *P*-values are based on 9999 permutations (i.e. the lowest possible *P*-value is 0.0001)

	Df	SS	*F*	*R^2^*	*P*
Tree species	1	0.8052	5.6484	0.09982	**0.0001**
Decay class	1	0.6076	4.2622	0.07532	**0.0001**
Management type	2	0.3967	1.3914	0.04918	0.0527
Tree species x Decay class	1	0.2377	1.6674	0.02947	**0.0315**
Tree species x Management type	2	0.4011	1.4068	0.04972	**0.0466**
Decay class x Management type	2	0.3474	1.2186	0.04307	0.1492
Tree species x Decay class x Management type	2	0.2815	0.9872	0.03489	0.4543
Residuals	35	4.9895		0.61853	
Total	46	8.0667		1	

**Table 2:** Goodness-of-fit statistics (*R*^2^) for parameters fitted to the non-metric multidimensional scaling (NMDS) ordination of bacterial community structure. The significance estimates were based on 999 permutations. Significant factors (Bonferroni corrected *P* < 0.05) are indicated in bold. Marginally significant variables (Bonferroni corrected *P* < 0.10) are indicated in italics. Data on remaining mass%, density, corrected N, and C content are auto-correlated (compare Table S2)

	*Fagus* and *Picea*		*Fagus*		*Picea*	
	*R^2^*	*P*	*R^2^*	*P*	*R^2^*	*P*
decay class	0.5257	**0.001**	0.6087	**0.001**	0.5205	**0.001**
remaining mass%	0.6449	**0.001**	0.7669	**0.001**	0.5999	**0.001**
volume (m^3^)	0.1519	**0.025**	0.3149	**0.027**	0.0806	0.308
wood density (g/cm^3^)	0.7144	**0.001**	0.7536	**0.001**	0.6133	**0.001**
rel. wood moisture%	0.2959	**0.001**	0.4438	**0.004**	0.3654	**0.013**
C%	0.3011	**0.001**	0.2357	**0.073**	0.1944	0.126
N%	0.1352	**0.043**	0.1989	0.103	0.204	0.112
C/N	0.1932	**0.008**	0.1917	0.109	0.2071	**0.09**
C (g/cm^3^)	0.7175	**0.001**	0.7569	**0.001**	0.6174	**0.001**
N (g/cm^3^)	0.4622	**0.001**	0.3728	**0.011**	0.1601	0.163
pH	0.493	**0.001**	0.3439	**0.015**	0.1438	0.197
