# Erratum: A pyrosequencing insight into sprawling bacterial diversity and community dynamics in decaying deadwood logs of *Fagus sylvatica* and *Picea abies*

**DOI:** 10.1038/srep10498

**Published:** 2016-05-10

**Authors:** Björn Hoppe, Dirk Krüger, Tiemo Kahl, Tobias Arnstadt, François Buscot, Jürgen Bauhus, Tesfaye Wubet

Scientific Reports 5: Article number: 945610.1038/srep09456; published online: 04082015; updated: 05102016

The original HTML version of this Article contained typographical errors in the spelling of the author Dirk Krüger which was incorrectly given as Krüger Krger. This has now been corrected.

Additionally, there was a typographical error in the Acknowledgements section:

“Christina Lachmann helped with bioinformatics.”

now reads:

“Christina Weißbecker helped with bioinformatics.”

