# Supplementary material for: A pyrosequencing insight into sprawling bacterial diversity and community dynamics in decaying deadwood logs of Fagus sylvatica and Picea abies
Source: Sci Rep. 2015 Apr 8;5:9456. doi: 10.1038/srep09456 (PMC4389208; doi:10.1038/srep09456)
Supplement: Supplementary Information [file srep09456-s1.pdf]

A pyrosequencing insight into sprawling bacterial diversity and community dynamics in decaying deadwood logs of *Fagus sylvatica* and *Picea abies*

Björn Hoppe*†‡, Dirk Krüger*, Tiemo Kahl†, Tobias Arnstadt¥, François Buscot*≠, Jürgen Bauhus†, Tesfaye Wubet*≠‡

Supplementary Information

Sampling

In April 2009, the initial experimental set-up comprised 48 logs (24 of *P. abies* and 24 of *F. sylvatica*) located on the forest floor of nine very intensively investigated 1 ha forest plots (VIPs) within the "Schwäbische Alb" exploratory, with three plots representing three different forest types and management regimes, respectively: (i) unmanaged beech forests, where timber harvesting stopped 2 - 7 decades ago, (ii) managed beech forests dominated by *Fagus sylvatica* and (iii) managed spruce forests dominated by *Picea abies,* which in both cases are characterized by uniform tree species composition, forest structure and site conditions, were randomly selected and characterized (Fig. S1, Table S1). The logs were selected to ensure that some of the *Fagus* logs were located in *Picea*-dominated plots and *vice versa*.

In June 2009, 3-7 wood samples were collected from each log. A minimum of 3 wood samples was taken for logs up to a length of 5 m, and additional wood samples were taken if the log was longer, to fully represent the entire log (compare Purahong *et al.* [1]). That number of required wood samples was determined according to the formula $x = 3 + \frac{y-5}{5}$ (with x = the rounded number of drills per log, and y = length of deadwood log). To take the samples, we used a cordless Makita BDF451 drill (Makita, Anja, Japan) equipped with a 2 x 42 cm wood auger as described by Hoppe *et al.* [2]. The upper surface layer and bark of the deadwood were removed to avoid contamination by bacteria from outside.

Each deadwood log was assigned to one of 4 decay classes based on its remaining mass (%) (which was determined upon mass and density loss) by k-means cluster analysis as described by Kahl *et al.* [3]. Higher decay classes corresponded to more extensive decay (Table S7). The estimated point of death of deadwood logs at the date of sampling varied between 3 and 27 years (based on dendro-chronological analyses) and death was due to various reasons, ranging from windbreaks to logging with leftover trees.

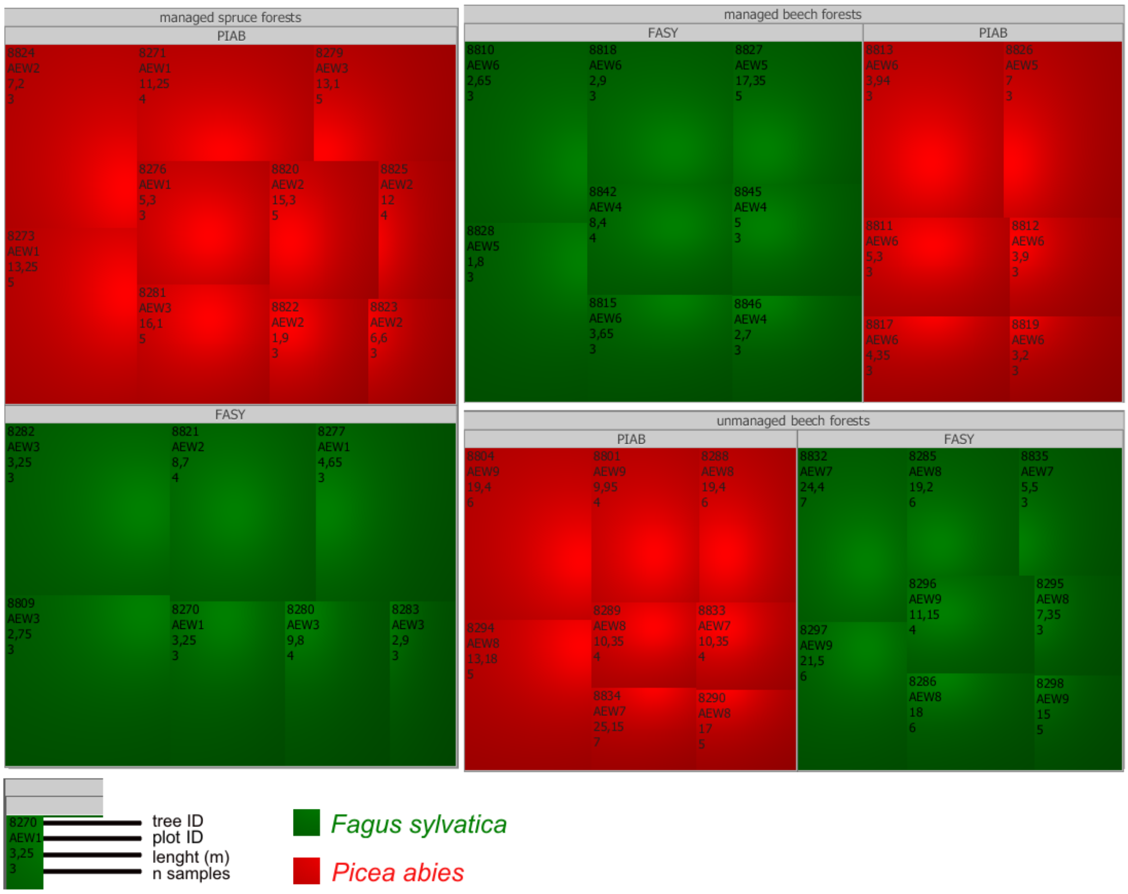

Fig. S1: Sampling scheme visualized using Treemap v. 3.1.0. (Macrofocus, Zurich, Switzerland) in squarified layout. Items are hierarchically grouped by management type and tree species. Treemap cell size is proportional to remaining mass in % (smaller cells = more decayed logs) Colors represent tree species (red = *Picea abies*, green = *Fagus sylvatica*). The labels in the cells indicate tree ID, plot ID, length of deadwood log in m and the number of wood subsamples taken per log, as shown in legend.

Table S1: Sampling design: Distribution of deadwood logs according to tree species and the respective 9 forest plots which were assigned to three different forest management types.

		Management type		
		Unmanaged beech forests (3 plots)	managed beech forests (3 plots)	managed spruce forests (3 plots)
Deadwood tree species	*Fagus sylvatica*	**8**	**8**	**8**
	Picea abies	**8**	**6**	**10**

Table S2a: Mean values of wood properties and standard error for each tree species related decay class and ANOVA *P* values. Differences between decay classes were analyzed by employing one-way analysis of variance and Tukey pair-wise comparisons. Significant ANOVA *P* values are shown in bold ($P < 0.05$). Different letters indicate differences among decay classes ($P < 0.05$).

	Decay class	C/N	C %	N %	C (g/cm^3)	N (g/cm^3)	Density (g/cm^3)	rel. wood moisture	pH
Fagus sylvatica	**1 (7 logs)**	376.88 ± 11.55 cd	48.37 ± 0.18 b	0.13 ± 0.005 bc	0.25 ± 0.01 a	0.0007 ± 0.00004 a	0.51 ± 0.02 a	52.2 ± 5.76 b	5.28 ± 0.12 a
	**2 (6 logs)**	324.02 ± 24.25 cd	47.92 ± 0.2 b	0.15 ± 0.013 bc	0.18 ± 0.01 b	0.0006 ± 0.00006 a	0.37 ± 0.01 b	114.21 ± 15.84 ab	4.84 ± 0.09 ab
	**3 (7 logs)**	282.87 ± 29.19 cd	47.52 ± 0.34 b	0.18 ± 0.015 ab	0.15 ± 0 bc	0.0005 ± 0.00004 a	0.31 ± 0.01 bc	138.22 ± 21.24 ab	5.12 ± 0.13 a
	**4 (3 logs)**	193.7 ± 15.62 d	47.51 ± 0.33 b	0.25 ± 0.02 a	0.09 ± 0.02 de	0.0005 ± 0.00009 ab	0.2 ± 0.03 de	155.15 ± 9.14 ab	4.99 ± 0.13 ab
Picea abies	**1 (5 logs)**	629.69 ± 48.44 a	49.29 ± 0.31 ab	0.08 ± 0.007 c	0.17 ± 0 bc	0.0003 ± 0.00003 bc	0.35 ± 0.01 b	48.72 ± 11.59 b	4.63 ± 0.1 abc
	**2 (5 logs)**	602.56 ± 19.24 ab	49.92 ± 0.22 ab	0.08 ± 0.003 c	0.13 ± 0 cd	0.0002 ± 0.00001 bc	0.27 ± 0.01 cd	65.45 ± 13.38 b	4.33 ± 0.03 bc
	**3 (5 logs)**	591.22 ± 38.75 ab	49.16 ± 0.35 ab	0.08 ± 0.005 c	0.11 ± 0.01 de	0.0002 ± 0.00001 c	0.22 ± 0.01 de	95.27 ± 8.91 ab	4.04 ± 0.16 bc
	**4 (9 logs)**	422.87 ± 52.44 bc	51.39 ± 0.94 ab	0.14 ± 0.022 bc	0.08 ± 0 e	0.0002 ± 0.00003 c	0.15 ± 0.01 e	163.06 ± 24.62 a	4.26 ± 0.15 c
p		<0.001	<0.002	<0.001	<0.001	<0.001	<0.001	<0.003	<0.001

Table S2b: Correlations among assessed wood physico-chemical properties. Significant values ($P < 0.05$) are given in bold.

	remaining mass in %	volume	density	rel. wood moisture	C %	N %	C/N	C (g/cm^3)	N (g/cm^3)	pH
remaining mass in %										
volume	-0.16									
density	**0.89**	-0.12								
rel. wood moisture %	**-0.67**	0.03	**-0.53**							
C %	-0.23	0.11	-0.39	0.25						
N %	-0.38	-0.05	-0.11	**0.6**	-0.32					
C/N	0.26	0.01	-0.09	**-0.53**	0.42	**-0.92**				
C (g/cm^3)	**0.9**	-0.13	**-0.99**	**-0.53**	-0.34	-0.14	-0.05			
N (g/cm^3)	0.42	-0.09	**0.73**	0.02	**-0.52**	**0.56**	**-0.69**	**0.7**		
pH	0.32	0.05	0.55	-0.03	**-0.5**	0.45	-0.6	**0.53**	**0.71**	

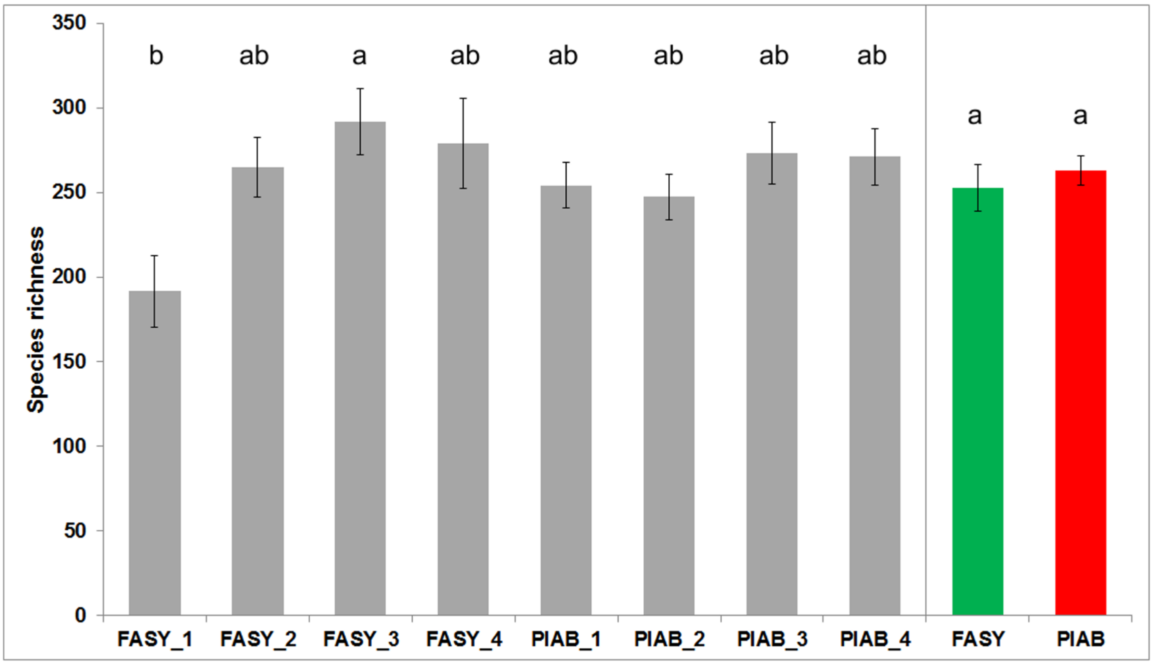

Fig. S2: Bacterial richness of the two deadwood species *Fagus sylvatica* and *Picea abies* of 4 different decay classes (e.g. FASY1 = *Fagus sylvatica*, decay class 1) at a genetic cutoff of 3%. Richness is displayed as the mean number of observed sequences per tree decay classes. Differences of bacterial species richness between decay classes and tree species were analyzed by employing one-way analysis of variance and Fisher's Least Significant Difference (LSD) *post hoc* test.

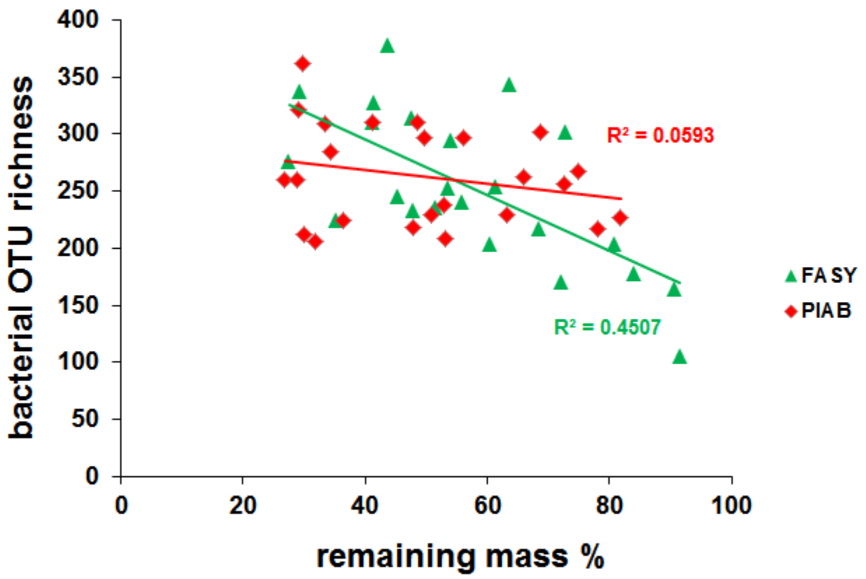

Fig. S3: Correlation between bacterial OTU richness and remaining mass in %. Green triangles represent data on FASY (*Fagus sylvatica*) and red diamonds on PIAB (*Picea abies*).

Table S3: Mean relative abundances of dominant bacterial phyla (including proteobacterial subphyla) at different decay stages. Differences of bacterial abundances between decay classes and tree species were analyzed by employing one-way analysis of variance and Fisher's Least Significant Difference (LSD) *post hoc* test.

	FASY 1		FASY 2		FASY 3		FASY 4		PIAB 1		PIAB 2		PIAB 3		PIAB 4		*P* - value
Alphaproteobacteria	28.1 ± 3	c	38.5 ± 1.8	abc	43.5 ± 2.3	ab	50.7 ± 1.9	a	30.4 ± 3.7	bc	45.4 ± 2	ab	42.2 ± 3	ab	44.1 ± 1.5	ab	<0.001
Acidobacteria	13.3 ± 3.4	a	16.5 ± 3.7	a	14 ± 3.7	a	15.1 ± 3.2	a	19.5 ± 0.7	a	20.4 ± 0.9	a	22.4 ± 1.9	a	20.7 ± 1.1	a	0.19
Actinobacteria	13.2 ± 1.7	a	11.5 ± 0.7	a	10 ± 1.1	a	6.4 ± 0.5	a	14.2 ± 1.8	a	10.8 ± 1	a	11.9 ± 1.3	a	10.2 ± 0.9	a	*0.08*
Gammaproteobacteria	12.7 ± 1.5	a	9.7 ± 2.2	ab	8.1 ± 0.4	ab	5.7 ± 1.8	ab	10.8 ± 3.8	ab	5.6 ± 1	ab	5.3 ± 1.1	b	6.8 ± 0.7	ab	0.047
Bacteroidetes	9.1 ± 2.5	a	5.9 ± 0.6	a	10.4 ± 1.7	a	8.7 ± 0.7	a	8.3 ± 1.5	a	5.9 ± 0.3	a	5.8 ± 0.6	a	5.5 ± 0.6	a	0.149
Betaproteobacteria	11.8 ± 2.8	a	7 ± 2.3	a	4.7 ± 0.8	a	7 ± 0.8	a	7.2 ± 1.1	a	7.6 ± 0.9	a	6.8 ± 1.6	a	5.2 ± 0.6	a	0.17
Firmicutes	9.6 ± 3.2	a	6.5 ± 1.6	ab	4.5 ± 1.6	ab	0.6 ± 0.2	b	5.2 ± 0.8	ab	1 ± 0.2	ab	0.9 ± 0.3	b	0.4 ± 0.2	b	0.003
Cyanobacteria	1.3 ± 0.3	a	2.2 ± 0.6	a	1.5 ± 0.3	a	3.1 ± 0.8	a	2.6 ± 1.1	a	1.3 ± 0.2	a	2 ± 0.1	a	2.5 ± 0.2	a	0.23
Deltaproteobacteria	0.6 ± 0.2	b	1.3 ± 0.5	b	1.9 ± 0.7	ab	1.7 ± 1	ab	1.2 ± 0.6	b	1.2 ± 0.6	b	1.6 ± 0.7	ab	2.6 ± 0.9	a	0.001

Table S4: Pearson rank correlation table of selected dominant bacterial phyla (including proteobacterial subphyla) with wood physico-chemical properties. Significant correlations ($P < 0.05$) are displayed in bold. Calculations were performed separately for the respective tree species.

	Fagus	*Picea*	*Fagus*	*Picea*	*Fagus*	*Picea*	*Fagus*	*Picea*
	C/N		remaining mass %		rel. wood moisture %		pH	
Alphaproteobacteria	**-0.44**	-0.03	**-0.75**	**-0.49**	**0.52**	0.24	0.05	**-0.4**
Acidobacteria	0.13	-0.02	-0.12	-0.1	0.2	0.07	**-0.46**	-0.13
Actinobacteria	0.27	0.31	**0.54**	**0.4**	**-0.42**	**-0.43**	0.26	0.33
Gammaproteobacteria	-0.03	-0.21	**0.52**	0.25	-0.33	0.15	0.21	0.39
Bacteroidetes	-0.05	0.02	0.02	**0.43**	-0.25	-0.37	0.28	0.18
Betaproteobacteria	0.12	0.19	0.39	0.27	-0.14	**-0.45**	0.06	-0.14
Firmicutes	**0.4**	0.3	**0.45**	**0.7**	-0.43	-0.35	0.04	**0.41**
Cyanobacteria	-0.14	-0.06	**-0.38**	-0.18	0.23	0.16	-0.27	-0.18
Deltaproteobacteria	**-0.41**	**-0.53**	**-0.64**	**-0.56**	**0.52**	**0.65**	0.04	0.21

Table S5: Pearson rank correlation table of selected methylo- and methanotrophic bacteria with wood physico-chemical properties. Significant correlations ($P < 0.05$) are displayed in bold.

Genus	C/N		remaining mass %		rel. wood moisture %		pH	
	Fagus	*Picea*	*Fagus*	*Picea*	*Fagus*	*Picea*	*Fagus*	*Picea*
Methylovirgula	**-0.38**	-0.17	**-0.7**	-0.24	**0.69**	-0.28	-0.23	-0.04
Methyloferula	-0.29	0.1	-0.31	-0.02	**0.56**	-0.01	-0.3	-0.06
Methylocella	0.18	-0.32	-0.02	-0.08	-0.23	-0.07	0.26	-0.1
Methylocystis	-0.02	-0.22	-0.06	-0.03	0.24	-0.33	-0.22	-0.29

Table S6: One-way analysis of similarity (ANOSIM) based on each two distance measures using relative abundance and presence/ absence data for different wood-inhabiting bacterial communities on two different tree species (*Fagus sylvativca* and *Picea abies*).

Tree species		Bray-Curtis		Euclidean	
		R	*P*	*R*	*P*
Fagus sylvatica vs. Picea abies	Rel. abundance	0.3098	**0.001**	0.327	**0.001**
		Jaccard		Sörenson	
		R	*P*	*R*	*P*
	Presence/ absence	0.3141	**0.001**	0.3141	**0.001**

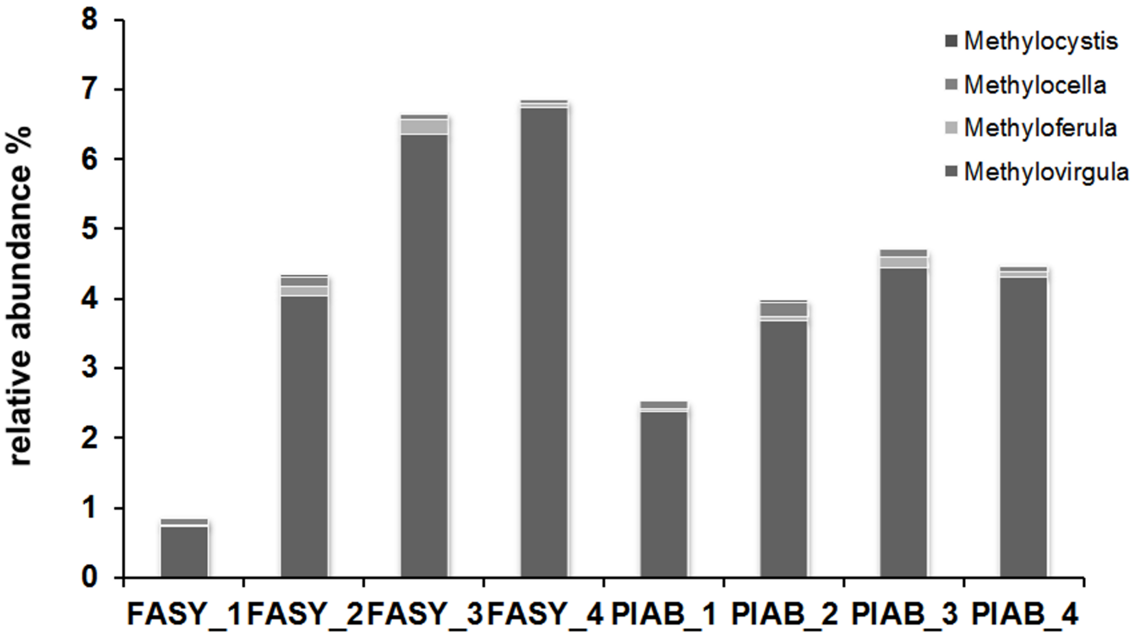

Fig. S4: Relative abundances of selected bacterial OTUs (methano- and methylotrophic genera) per wood tree species (*Fagus sylvatica* = FASY, *Picea abies* = PIAB) and according decay classes (1-4).

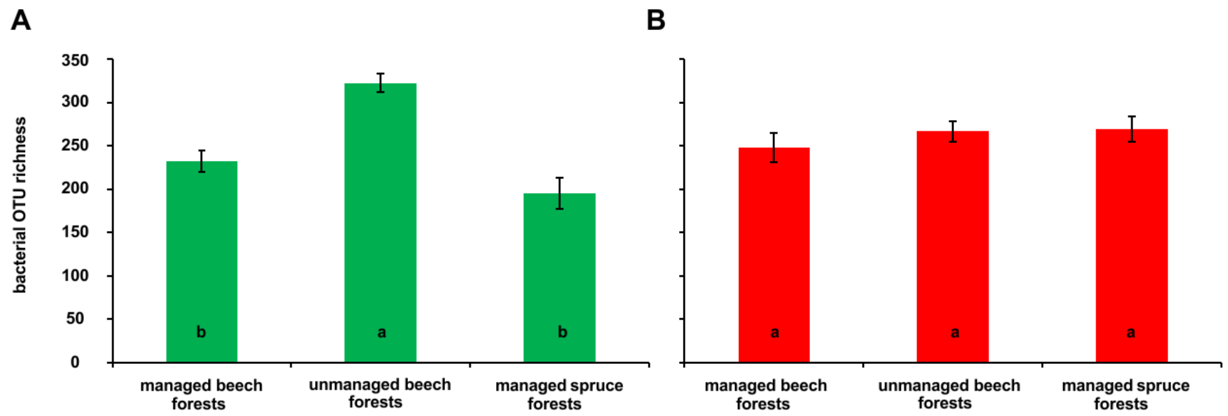

Fig. S5: Mean bacterial OTU richness in differently managed forest types (including all decay classes). (A) *Fagus sylvatica* logs in each of the three differently managed forest plots and (B) *vice versa* of *Picea abies* logs. Fisher's Least Significant Difference (LSD) *post hoc* test were calculated separately for (A) and (B).

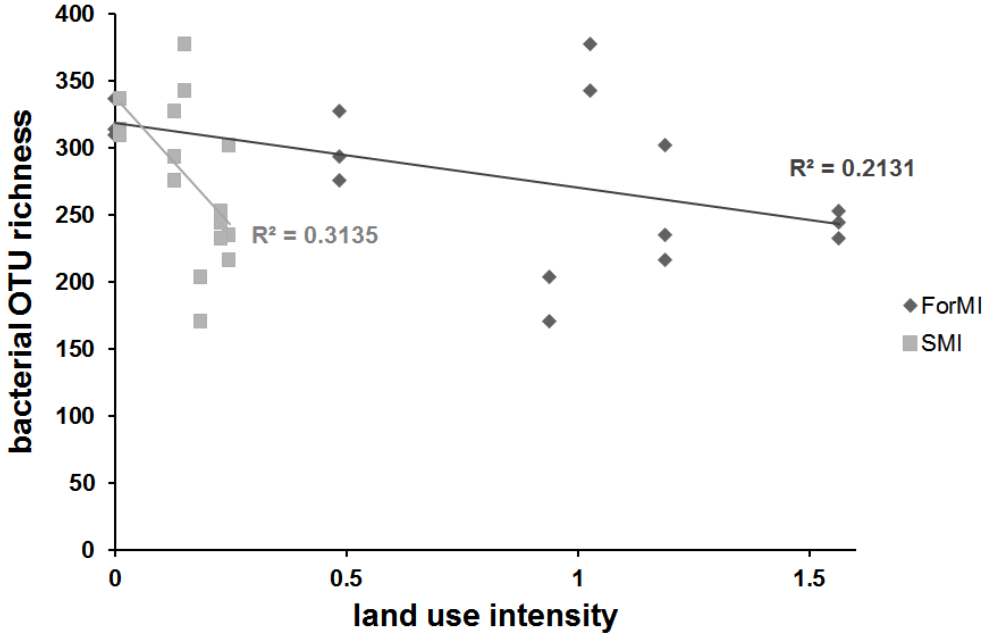

Fig. S6: Correlation between bacterial OTU richness on deadwood logs of *Fagus sylvatica* and the two land use intensity indices ForMI (light grey squares) and SMI (dark grey diamonds).

Bacterial family (BF)	Average BF dissimilarity	Contribution of BF to group dissimilarity	**FASY 1**	**FASY 2**	**FASY 3**	**FASY 4**	Bacterial family (BF)	Average family dissimilarity	Contribution of BF to group dissimilarity	**PIAB 1**	**PIAB 2**	**PIAB 3**	**PIAB 4**
Overall dissimilarity avg. 40.13			Mean abundance				Overall dissimilarity avg. 29.75			Mean abundance			
Acidobacteriaceae	6.7	16.7	12.5	13.7	13.6	13.6	*Acetobacteraceae*	6.0	20.3	11.1	20.5	17.8	14.3
Burkholderiaceae	3.7	9.1	7.5	5.2	2.7	4.9	*Acidobacteriaceae*	3.1	10.3	18.5	21.2	19.6	15.9
Beijerinckiaceae	3.4	8.5	1.5	5.1	8.1	7.9	*Burkholderiaceae*	2.1	7.1	5.3	5.3	5.4	3.5
Acetobacteraceae	3.1	7.7	5.6	5.7	6.0	13.6	*Beijerinckiaceae*	2.0	6.6	4.0	5.4	5.5	5.6
Hyphomicrobiaceae	2.9	7.1	2.6	6.0	8.6	5.4	*Xanthobacteraceae*	1.7	5.8	0.8	0.8	1.0	4.1
Sphingobacteriaceae	2.3	5.8	6.4	2.8	4.1	3.9	*Microbacteriaceae*	1.3	4.5	3.6	2.0	2.7	0.9
Xanthomonadaceae	2.2	5.4	4.4	3.6	0.9	0.3	*Sinobacteraceae*	1.3	4.5	1.2	1.9	2.1	4.1
Microbacteriaceae	2.0	5.0	5.6	4.0	3.7	1.5	*Hyphomicrobiaceae*	1.2	4.1	2.4	2.4	3.4	3.4
Veillonellaceae	2.0	4.9	2.8	3.2	0.7	0.1	*Veillonellaceae*	1.2	3.9	3.1	0.2	0.9	0.1
Bradyrhizobiaceae	1.9	4.6	4.4	5.5	4.7	4.2	*Rhodospirillaceae*	1.1	3.8	1.8	3.1	3.5	3.1
Rhodospirillaceae	1.8	4.6	0.8	2.5	3.5	5.9	*Bradyrhizobiaceae*	1.1	3.7	3.5	1.9	3.3	2.8
Sphingomonadaceae	1.8	4.5	4.0	3.6	2.1	3.0	*Chitinophagaceae*	1.1	3.6	3.5	3.0	2.7	2.9
Chitinophagaceae	1.4	3.6	1.7	2.6	4.1	4.1	*Frankiaceae*	1.0	3.4	2.7	1.7	1.5	1.2
Caulobacteraceae	1.2	3.0	1.5	1.8	3.4	4.6	*Sphingobacteriaceae*	1.0	3.3	3.9	3.4	2.0	2.1
Comamonadaceae	1.0	2.6	3.2	1.5	1.4	1.5	*Caulobacteraceae*	1.0	3.3	3.3	3.2	3.5	3.6
Sinobacteraceae	1.0	2.4	1.1	1.3	2.5	2.0	*Acidobacteria I.S.*	0.9	3.1	0.8	0.5	0.9	2.1
Xanthobacteraceae	0.7	1.7	1.2	1.3	1.4	0.7	*Sphingomonadaceae*	0.8	2.8	1.3	1.1	2.4	1.2
Frankiaceae	0.4	1.0	1.2	1.1	0.8	0.5	*Acidothermaceae*	0.6	2.2	0.5	0.8	0.7	1.8
Acidothermaceae	0.4	0.9	0.4	0.4	0.7	0.2	*Comamonadaceae*	0.6	2.0	1.7	0.8	0.9	0.7
Acidobacteria I.S.	0.4	0.9	0.5	0.8	0.7	0.2	*Xanthomonadaceae*	0.6	1.9	1.4	1.2	0.8	0.9

Fig. S7: Relative abundances and their contribution to total community variation of bacterial families which represent >0.75 of the sequences of the whole dataset as calculated by SIMPER. Both *Fagus* (FASY) and *Picea* (PIAB) were separated in four wood decay classes. Heatmaps (white = zero, red = maximum abundance) were overlaid separately for *Fagus* and *Picea*. I.S. = *insertae sedis*.

Table S7: List of MID tags for deadwood logs (samples) and associated plot metadata.

CWD ID	MID tag used in pyrosequencing	no. of seqs.	norm-alized no. of seqs.	tree species	rem. mass %	de-cay class	forest manag.	plot ID	Gauss-Krüger coordinates		coordinates		alti-tude (m)	soil type
									RW	HW	latitude	longitude		
8270	ACGAGTGCGT	2288	1846	*F. sylvatica*	61.4	2	managed spruce forests	AEW1	3524799	5371201	48.478064°N	9.334397°E	763	Cambisol
8271	ACGCTCGACA	2753	1846	*P. abies*	66	2	managed spruce forests	AEW1	3524799	5371201	48.478064°N	9.334397°E	763	Cambisol
8273	AGACGCACTC	2501	1846	*P. abies*	75	1	managed spruce forests	AEW1	3524799	5371201	48.478064°N	9.334397°E	763	Cambisol
8276	AGCACTGTAG	2851	1846	*P. abies*	53	2	managed spruce forests	AEW1	3524799	5371201	48.478064°N	9.334397°E	763	Cambisol
8277	ATCAGACACG	1976	1846	*F. sylvatica*	80.8	1	managed spruce forests	AEW1	3524799	5371201	48.478064°N	9.334397°E	763	Cambisol
8820	ATATCGCGAG	2740	1846	*P. abies*	48.5	3	managed spruce forests	AEW2	3526110	5360300	48.37998°N	9.351452°E	749	Leptosol
8821	CGTGTCTCTA	2693	1846	*F. sylvatica*	84	1	managed spruce forests	AEW2	3526110	5360300	48.37998°N	9.351452°E	749	Leptosol
8822	CTCGCGTGTC	2644	1846	*P. abies*	33.5	4	managed spruce forests	AEW2	3526110	5360300	48.37998°N	9.351452°E	749	Leptosol
8823	TAGTATCAGC	1933	1846	*P. abies*	29.8	4	managed spruce forests	AEW2	3526110	5360300	48.37998°N	9.351452°E	749	Leptosol
8824	TCTCTATGCG	2150	1846	*P. abies*	78.1	1	managed spruce forests	AEW2	3526110	5360300	48.37998°N	9.351452°E	749	Leptosol
8825	TGATACGTCT	2049	1846	*P. abies*	34.4	4	managed spruce forests	AEW2	3526110	5360300	48.37998°N	9.351452°E	749	Leptosol
8279	TACTGAGCTA	2295	1846	*P. abies*	53.1	2	managed spruce forests	AEW3	3526400	5363890	48.412253°N	9.355592°E	715	Cambisol
8280	CATAGTAGTG	2503	1846	*F. sylvatica*	55.8	2	managed spruce forests	AEW3	3526400	5363890	48.412253°N	9.355592°E	715	Cambisol
8281	CGAGAGATAC	2292	1846	*P. abies*	50.8	3	managed spruce forests	AEW3	3526400	5363890	48.412253°N	9.355592°E	715	Cambisol
8282	ATACGACGTA	1893	1846	*F. sylvatica*	91.5	1	managed spruce forests	AEW3	3526400	5363890	48.412253°N	9.355592°E	715	Cambisol
8283	TCACGTACTA	2331	1846	*F. sylvatica*	35.3	4	managed spruce forests	AEW3	3526400	5363890	48.412253°N	9.355592°E	715	Cambisol
8809	TCTACGTAGC	2535	1846	*F. sylvatica*	90.6	1	managed spruce forests	AEW3	3526400	5363890	48.412253°N	9.355592°E	715	Cambisol
8842	TGTACTACTC	2183	1846	*F. sylvatica*	53.5	2	managed beech forests	AEW4	3518205	5362394	48.399088°N	9.244828°E	783	Cambisol
8845	ACGACTACAG	1864	1846	*F. sylvatica*	47.9	3	managed beech forests	AEW4	3518205	5362394	48.399088°N	9.244828°E	783	Cambisol
8846	CGTAGACTAG	2318	1846	*F. sylvatica*	45.5	3	managed beech forests	AEW4	3518205	5362394	48.399088°N	9.244828°E	783	Cambisol

CWD ID	MID tag used in pyrosequencing	no. of seqs.	norm-alized no. of seqs.	tree species	rem. mass %	de-cay class	forest manag.	plot ID	Gauss-Krüger coordinates		coordinates		alti-tude (m)	soil type
									RW	HW	latitude	longitude		
8826	TACGAGTATG	2317	1846	*P. abies*	68.7	1	managed beech forests	AEW5	3530 770	5364 730	48.419609°N	9.414683°E	809	Cambisol
8827	TACTCTCGTG	2538	1846	*F. sylvatica*	60.4	2	managed beech forests	AEW5	3530 770	5364 730	48.419609°N	9.414683°E	809	Cambisol
8828	TAGAGACGAG	2529	1846	*F. sylvatica*	72.2	1	managed beech forests	AEW5	3530 770	5364 730	48.419609°N	9.414683°E	809	Cambisol
8810	TCGTCGCTCG	2853	1846	*F. sylvatica*	72.9	1	managed beech forests	AEW6	3533 100	5361 900	48.394042°N	9.445939°E	767	Cambisol
8811	ACATACGCGT	2560	1846	*P. abies*	48	3	managed beech forests	AEW6	3533 100	5361 900	48.394042°N	9.445939°E	767	Cambisol
8812	ACGCGAGTAT	1990	1846	*P. abies*	36.5	4	managed beech forests	AEW6	3533 100	5361 900	48.394042°N	9.445939°E	767	Cambisol
8813	ACTACTATGT	2164	1846	*P. abies*	81.8	1	managed beech forests	AEW6	3533 100	5361 900	48.394042°N	9.445939°E	767	Cambisol
8815	ACTGTACAGT	2545	1846	*F. sylvatica*	51.5	3	managed beech forests	AEW6	3533 100	5361 900	48.394042°N	9.445939°E	767	Cambisol
8817	AGACTATACT	2754	1846	*P. abies*	41.3	3	managed beech forests	AEW6	3533 100	5361 900	48.394042°N	9.445939°E	767	Cambisol
8818	AGCGTCGTCT	2500	1846	*F. sylvatica*	68.5	1	managed beech forests	AEW6	3533 100	5361 900	48.394042°N	9.445939°E	767	Cambisol
8819	AGTACGCTAT	2257	1846	*P. abies*	31.7	4	managed beech forests	AEW6	3533 100	5361 900	48.394042°N	9.445939°E	767	Cambisol
8832	ATAGAGTACT	3039	1846	*F. sylvatica*	63.7	2	managed beech forests	AEW7	3519 430	5362 080	48.396228°N	9.261357°E	773	Leptosol
8833	CACGCTACGT	2520	1846	*P. abies*	28.9	4	managed beech forests	AEW7	3519 430	5362 080	48.396228°N	9.261357°E	773	Leptosol
8834	CAGTAGACGT	2049	1846	*P. abies*	29	4	managed beech forests	AEW7	3519 430	5362 080	48.396228°N	9.261357°E	773	Leptosol
8835	CGACGTGACT	2385	1846	*F. sylvatica*	43.8	3	managed beech forests	AEW7	3519 430	5362 080	48.396228°N	9.261357°E	773	Leptosol
8285	TACACACACT	2907	1846	*F. sylvatica*	47.6	3	unmanaged beech forestst	AEW8	3528 400	5360 600	48.38258°N	9.382386°E	779	Cambisol
8286	TACACGTGAT	1989	1846	*F. sylvatica*	41.2	3	unmanaged beech forestst	AEW8	3528 400	5360 600	48.38258°N	9.382386°E	779	Cambisol
8288	TACAGATCGT	2839	1846	*P. abies*	49.7	3	unmanaged beech forestst	AEW8	3528 400	5360 600	48.38258°N	9.382386°E	779	Cambisol
8289	TACGCTGTCT	2712	1846	*P. abies*	30	4	unmanaged beech forestst	AEW8	3528 400	5360 600	48.38258°N	9.382386°E	779	Cambisol
8290	TAGTGTAGAT	2730	1846	*P. abies*	26.9	4	unmanaged beech	AEW8	3528 400	5360 600	48.38258°N	9.382386°E	779	Cambisol

CWD ID	MID tag used in pyrosequencing	no. of seqs.	norm-alized no. of seqs.	tree species	rem. mass %	de-cay class	forest manag.	plot ID	Gauss-Krüger coordinates		coordinates		alti-tude (m)	soil type
									RW	HW	latitude	longitude		
							foreststst							
8294	**TCGATCACGT**	2377	1846	*P. abies*	63.2	2	unmanaged beech foreststst	AEW8	3528 400	5360 600	48.38258°N	9.382386°E	779	Cambisol
8295	**TCGCACTAGT**	2659	1846	*F. sylvatica*	29.4	4	unmanaged beech foreststst	AEW8	3528 400	5360 600	48.38258°N	9.382386°E	779	Cambisol
8296	**TCTAGCGACT**	2541	1846	*F. sylvatica*	41.5	3	unmanaged beech foreststst	AEW9	3530 840	5359 140	48.369336°N	9.41522°E	753	Leptosol
8297	**TCTATACTAT**	2733	1846	*F. sylvatica*	54.1	2	unmanaged beech foreststst	AEW9	3530 840	5359 140	48.369336°N	9.41522°E	753	Leptosol
8298	**TGACGTATGT**	3132	1846	*F. sylvatica*	27.6	4	unmanaged beech foreststst	AEW9	3530 840	5359 140	48.369336°N	9.41522°E	753	Leptosol
8801	**TGTGAGTAGT**	2335	1846	*P. abies*	56	2	unmanaged beech foreststst	AEW9	3530 840	5359 140	48.369336°N	9.41522°E	753	Leptosol
8804	**ACAGTATATA**	3004	1846	*P. abies*	72.7	1	unmanaged beech foreststst	AEW9	3530 840	5359 140	48.369336°N	9.41522°E	753	Leptosol

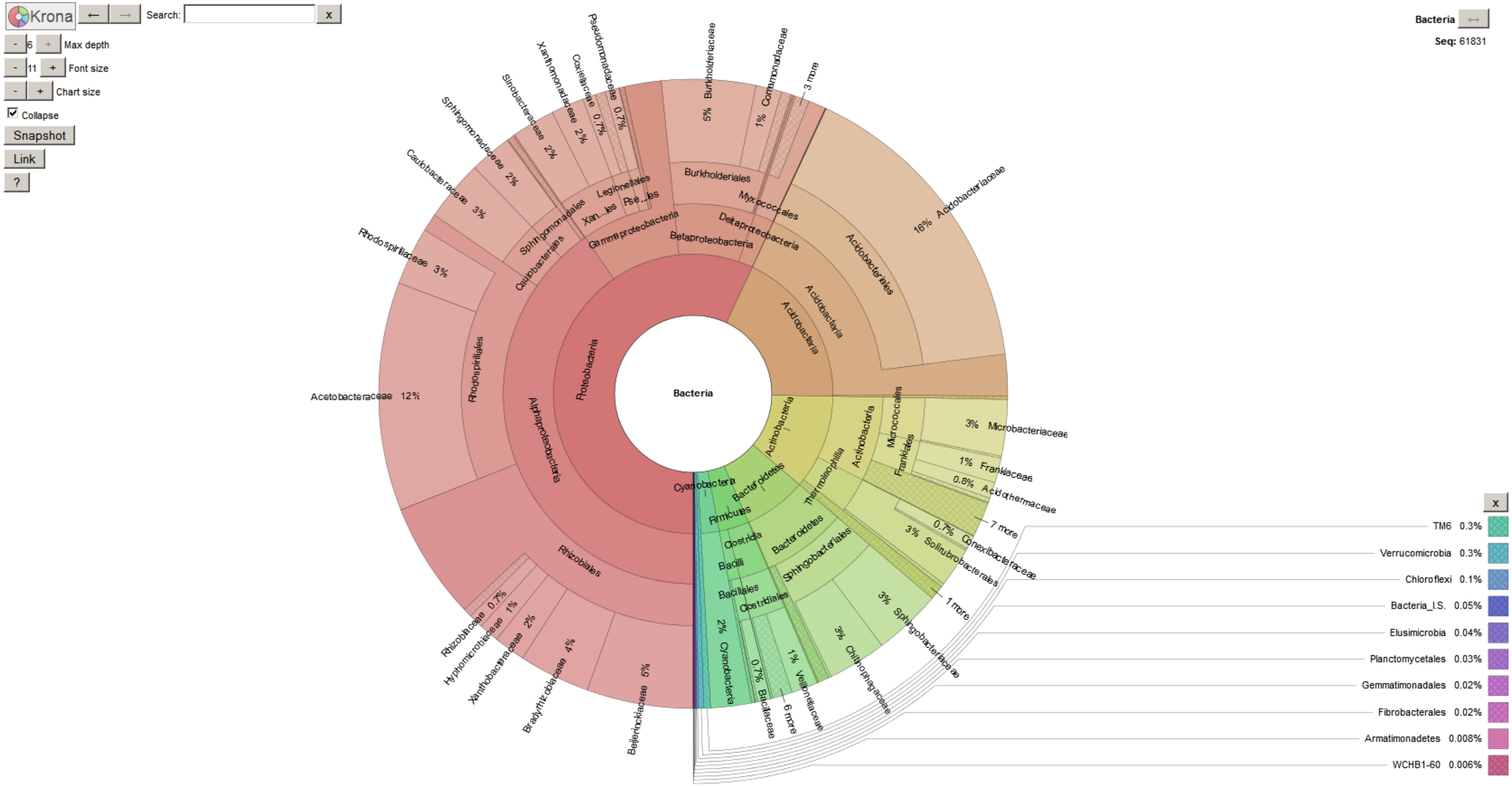

Kronafile SK1: Interactive web-visualization of taxonomic information on all deadwood logs using Krona (http://sourceforge.net/projects/krona/; Ondov *et al.* 2011 [4]).

Instruction to convert into .html

-> please copy the complete syntax (.html source code) into any text editor and save as “Kronafile SK1.html”
-> the file then opens by double click in your favorite webbrowser

```
<!DOCTYPE html PUBLIC "-//W3C//DTD XHTML 1.0 Strict//EN" "http://www.w3.org/TR/xhtml1/DTD/xhtml1-strict.dtd">
<html xmlns="http://www.w3.org/1999/xhtml" xml:lang="en" lang="en">
    <head>
        <meta charset="utf-8"/>
        <link rel="shortcut icon" href="http://krona.sourceforge.net/img/favicon.ico"/>
        <script id="notfound">window.onload=function(){document.body.innerHTML="Could not get resources from \"http://krona.sourceforge.net\"."}</script>
        <script src="http://krona.sourceforge.net/src/krona-2.0.js"></script>
    </head>
    <body>
        <img id="hiddenImage" src="http://krona.sourceforge.net/img/hidden.png"  style="display:none"/>
        <noscript>Javascript must be enabled to view this page.</noscript>
        <div style="display:none"><krona>
            <attributes magnitude="magnitude">
                <attribute display="Seq">magnitude</attribute>
            </attributes>
<node name="Bact"><magnitude><val> 61831</val></magnitude>
<node name="Bacteria"><magnitude><val> 61831</val></magnitude>
<node name="Proteobacteria"><magnitude><val> 35207</val></magnitude>
<node name="Alphaproteobacteria"><magnitude><val> 24969</val></magnitude>
<node name="Alphaproteobacteria"><magnitude><val> 24969</val></magnitude>
<node name="Rhizobiales"><magnitude><val> 11739</val></magnitude>
<node name="Beijerinckiaceae"><magnitude><val> 3378</val></magnitude>
</node>
<node name="Bradyrhizobiaceae"><magnitude><val> 2263</val></magnitude>
</node>
<node name="Xanthobacteraceae"><magnitude><val> 1005</val></magnitude>
</node>
<node name="Hyphomicrobiaceae"><magnitude><val> 636</val></magnitude>
</node>
<node name="Rhizobiaceae"><magnitude><val> 461</val></magnitude>
</node>
<node name="Phyllobacteriaceae"><magnitude><val> 112</val></magnitude>
</node>
<node name="Methylocystaceae"><magnitude><val> 77</val></magnitude>
</node>
<node name="Methylobacteriaceae"><magnitude><val> 67</val></magnitude>
</node>
<node name="Rhodobiaceae"><magnitude><val> 13</val></magnitude>
</node>
<node name="Brucellaceae"><magnitude><val> 8</val></magnitude>
</node>
</node>
<node name="Rhodospirillales"><magnitude><val> 9670</val></magnitude>
<node name="Acetobacteraceae"><magnitude><val> 7265</val></magnitude>
</node>
<node name="Rhodospirillaceae"><magnitude><val> 1845</val></magnitude>
</node>
</node>
<node name="Caulobacterales"><magnitude><val> 1944</val></magnitude>
<node name="Caulobacteraceae"><magnitude><val> 1944</val></magnitude>
</node>
</node>
<node name="Sphingomonadales"><magnitude><val> 1363</val></magnitude>
<node name="Sphingomonadaceae"><magnitude><val> 1329</val></magnitude>
</node>
<node name="Erythrobacteraceae"><magnitude><val> 34</val></magnitude>
</node>
</node>
<node name="Rickettsiales"><magnitude><val> 194</val></magnitude>
<node name="Rickettsiaceae"><magnitude><val> 25</val></magnitude>
</node>
</node>
<node name="Rhodobacterales"><magnitude><val> 9</val></magnitude>
<node name="Rhodobacteraceae"><magnitude><val> 9</val></magnitude>
</node>
</node>
</node>
</node>
<node name="Betaproteobacteria"><magnitude><val> 4262</val></magnitude>
<node name="Betaproteobacteria"><magnitude><val> 4262</val></magnitude>
<node name="Burkholderiales"><magnitude><val> 4125</val></magnitude>
<node name="Burkholderiaceae"><magnitude><val> 2987</val></magnitude>
</node>
<node name="Comamonadaceae"><magnitude><val> 822</val></magnitude>
</node>
<node name="Oxalobacteraceae"><magnitude><val> 258</val></magnitude>
</node>
<node name="Alcaligenaceae"><magnitude><val> 58</val></magnitude>
</node>
```

</node>
<node name="Nitrosomonadales"><magnitude><val> 54</val></magnitude>
<node name="Nitrosomonadaceae"><magnitude><val> 47</val></magnitude>
</node>
<node name="Gallionellaceae"><magnitude><val> 7</val></magnitude>
</node>
</node>
<node name="Neisseriales"><magnitude><val> 7</val></magnitude>
<node name="Neisseriaceae"><magnitude><val> 7</val></magnitude>
</node>
</node>
<node name="Rhodocyclales"><magnitude><val> 4</val></magnitude>
<node name="Rhodocyclaceae"><magnitude><val> 4</val></magnitude>
</node>
</node>
</node>
</node>
<node name="Gammaproteobacteria"><magnitude><val> 4942</val></magnitude>
<node name="Gammaproteobacteria"><magnitude><val> 4942</val></magnitude>
<node name="Xanthomonadales"><magnitude><val> 2414</val></magnitude>
<node name="Sinobacteraceae"><magnitude><val> 1374</val></magnitude>
</node>
<node name="Xanthomonadaceae"><magnitude><val> 1040</val></magnitude>
</node>
</node>
<node name="Pseudomonadales"><magnitude><val> 464</val></magnitude>
<node name="Pseudomonadaceae"><magnitude><val> 439</val></magnitude>
</node>
<node name="Moraxellaceae"><magnitude><val> 25</val></magnitude>
</node>
</node>
<node name="Legionellales"><magnitude><val> 739</val></magnitude>
<node name="Legionellaceae"><magnitude><val> 321</val></magnitude>
</node>
<node name="Coxiellaceae"><magnitude><val> 418</val></magnitude>
</node>
</node>
<node name="Oceanospirillales"><magnitude><val> 92</val></magnitude>
<node name="Halomonadaceae"><magnitude><val> 88</val></magnitude>
</node>
<node name="Oceanospirillaceae"><magnitude><val> 4</val></magnitude>
</node>
</node>
<node name="Enterobacteriales"><magnitude><val> 59</val></magnitude>
<node name="Enterobacteriaceae"><magnitude><val> 59</val></magnitude>
</node>
</node>
</node>
</node>
<node name="Deltaproteobacteria"><magnitude><val> 1030</val></magnitude>
<node name="Deltaproteobacteria"><magnitude><val> 1030</val></magnitude>
<node name="Myxococcales"><magnitude><val> 1003</val></magnitude>
<node name="Polyangiaceae"><magnitude><val> 257</val></magnitude>
</node>
<node name="Haliangiaceae"><magnitude><val> 163</val></magnitude>
</node>
<node name="Cystobacteraceae"><magnitude><val> 37</val></magnitude>
</node>
</node>
<node name="Desulfuromonadales"><magnitude><val> 23</val></magnitude>
</node>
</node>
</node>
</node>
<node name="Acidobacteria"><magnitude><val> 11346</val></magnitude>
<node name="Acidobacteria"><magnitude><val> 11235</val></magnitude>
<node name="Acidobacteria"><magnitude><val> 11235</val></magnitude>
<node name="Acidobacteriales"><magnitude><val> 9911</val></magnitude>
<node name="Acidobacteriaceae"><magnitude><val> 9911</val></magnitude>
</node>
</node>
</node>
</node>
<node name="Holophagae"><magnitude><val> 111</val></magnitude>
<node name="Holophagae"><magnitude><val> 111</val></magnitude>
<node name="Holophagales"><magnitude><val> 41</val></magnitude>
<node name="Holophagaceae"><magnitude><val> 41</val></magnitude>
</node>
</node>
</node>
</node>
</node>
<node name="Bacteroidetes"><magnitude><val> 4505</val></magnitude>
<node name="Bacteroidetes"><magnitude><val> 4459</val></magnitude>
<node name="Sphingobacteriia"><magnitude><val> 4106</val></magnitude>
<node name="Sphingobacteriales"><magnitude><val> 4106</val></magnitude>
<node name="Sphingobacteriaceae"><magnitude><val> 2161</val></magnitude>
</node>
<node name="Chitinophagaceae"><magnitude><val> 1868</val></magnitude>

```
</node>
</node>
</node>
<node name="Flavobacteria"><magnitude><val> 130</val></magnitude>
<node name="Flavobacteriales"><magnitude><val> 130</val></magnitude>
<node name="Flavobacteriaceae"><magnitude><val> 130</val></magnitude>
</node>
</node>
</node>
<node name="Bacteroidia"><magnitude><val> 83</val></magnitude>
<node name="Bacteroidales"><magnitude><val> 83</val></magnitude>
<node name="Porphyromonadaceae"><magnitude><val> 51</val></magnitude>
</node>
<node name="Bacteroidaceae"><magnitude><val> 32</val></magnitude>
</node>
</node>
</node>
<node name="Cytophagia"><magnitude><val> 140</val></magnitude>
<node name="Cytophagales"><magnitude><val> 140</val></magnitude>
<node name="Cytophagaceae"><magnitude><val> 122</val></magnitude>
</node>
<node name="Flammeovirgaceae"><magnitude><val> 18</val></magnitude>
</node>
</node>
</node>
</node>
</node>
<node name="Actinobacteria"><magnitude><val> 6784</val></magnitude>
<node name="Actinobacteria"><magnitude><val> 6784</val></magnitude>
<node name="Actinobacteria"><magnitude><val> 4450</val></magnitude>
<node name="Micrococcales"><magnitude><val> 1855</val></magnitude>
<node name="Microbacteriaceae"><magnitude><val> 1787</val></magnitude>
</node>
<node name="Cellulomonadaceae"><magnitude><val> 62</val></magnitude>
</node>
<node name="Intrasporangiaceae"><magnitude><val> 6</val></magnitude>
</node>
</node>
<node name="Frankiales"><magnitude><val> 1467</val></magnitude>
<node name="Acidothermaceae"><magnitude><val> 513</val></magnitude>
</node>
<node name="Frankiaceae"><magnitude><val> 785</val></magnitude>
</node>
<node name="Sporichthyaceae"><magnitude><val> 67</val></magnitude>
</node>
<node name="Nakamurellaceae"><magnitude><val> 69</val></magnitude>
</node>
</node>
<node name="Corynebacteriales"><magnitude><val> 322</val></magnitude>
<node name="Mycobacteriaceae"><magnitude><val> 295</val></magnitude>
</node>
<node name="Nocardiaceae"><magnitude><val> 27</val></magnitude>
</node>
</node>
<node name="Propionibacteriales"><magnitude><val> 442</val></magnitude>
<node name="Nocardioidaceae"><magnitude><val> 352</val></magnitude>
</node>
<node name="Propionibacteriaceae"><magnitude><val> 86</val></magnitude>
</node>
</node>
<node name="Streptomycetales"><magnitude><val> 112</val></magnitude>
<node name="Streptomycetaceae"><magnitude><val> 112</val></magnitude>
</node>
</node>
<node name="Kineosporiales"><magnitude><val> 78</val></magnitude>
<node name="Kineosporiaceae"><magnitude><val> 78</val></magnitude>
</node>
</node>
<node name="Micromonosporales"><magnitude><val> 128</val></magnitude>
<node name="Micromonosporaceae"><magnitude><val> 128</val></magnitude>
</node>
</node>
<node name="Pseudonocardiales"><magnitude><val> 23</val></magnitude>
<node name="Pseudonocardiaceae"><magnitude><val> 23</val></magnitude>
</node>
</node>
<node name="Streptosporangiales"><magnitude><val> 6</val></magnitude>
<node name="Streptosporangiaceae"><magnitude><val> 6</val></magnitude>
</node>
</node>
</node>
<node name="Thermoleophilia"><magnitude><val> 1872</val></magnitude>
<node name="Solirubrobacterales"><magnitude><val> 1755</val></magnitude>
<node name="Conexibacteraceae"><magnitude><val> 431</val></magnitude>
</node>
<node name="Solirubrobacteraceae"><magnitude><val> 24</val></magnitude>
</node>
<node name="Patulibacteraceae"><magnitude><val> 42</val></magnitude>
</node>
```

</node>
<node name="Gaiellales"><magnitude><val> 117</val></magnitude>
</node>
</node>
<node name="Acidimicrobiia"><magnitude><val> 462</val></magnitude>
<node name="Acidimicrobiales"><magnitude><val> 462</val></magnitude>
<node name="Iamiaceae"><magnitude><val> 11</val></magnitude>
</node>
</node>
</node>
</node>
</node>
<node name="Firmicutes"><magnitude><val> 2150</val></magnitude>
<node name="Firmicutes"><magnitude><val> 2150</val></magnitude>
<node name="Clostridia"><magnitude><val> 1538</val></magnitude>
<node name="Clostridiales"><magnitude><val> 1530</val></magnitude>
<node name="Veillonellaceae"><magnitude><val> 839</val></magnitude>
</node>
<node name="Lachnospiraceae"><magnitude><val> 154</val></magnitude>
</node>
<node name="Ruminococcaceae"><magnitude><val> 373</val></magnitude>
</node>
<node name="Clostridiaceae"><magnitude><val> 67</val></magnitude>
</node>
<node name="Peptococcaceae"><magnitude><val> 17</val></magnitude>
</node>
<node name="Christensenellaceae"><magnitude><val> 8</val></magnitude>
</node>
<node name="Eubacteriaceae"><magnitude><val> 5</val></magnitude>
</node>
</node>
<node name="Thermoanaerobacterales"><magnitude><val> 8</val></magnitude>
<node name="Thermoanaerobacteraceae"><magnitude><val> 8</val></magnitude>
</node>
</node>
</node>
<node name="Bacilli"><magnitude><val> 612</val></magnitude>
<node name="Bacillales"><magnitude><val> 495</val></magnitude>
<node name="Bacillaceae"><magnitude><val> 416</val></magnitude>
</node>
<node name="Paenibacillaceae"><magnitude><val> 50</val></magnitude>
</node>
<node name="Alicyclobacillaceae"><magnitude><val> 12</val></magnitude>
</node>
<node name="Staphylococcaceae"><magnitude><val> 6</val></magnitude>
</node>
<node name="Sporolactobacillaceae"><magnitude><val> 4</val></magnitude>
</node>
</node>
<node name="Lactobacillales"><magnitude><val> 117</val></magnitude>
<node name="Streptococcaceae"><magnitude><val> 113</val></magnitude>
</node>
</node>
</node>
</node>
</node>
<node name="Cyanobacteria"><magnitude><val> 1294</val></magnitude>
<node name="Cyanobacteria"><magnitude><val> 1294</val></magnitude>
</node>
</node>
<node name="TM6"><magnitude><val> 208</val></magnitude>
<node name="TM6"><magnitude><val> 208</val></magnitude>
</node>
</node>
<node name="Verrucomicrobia"><magnitude><val> 170</val></magnitude>
<node name="Verrucomicrobia"><magnitude><val> 56</val></magnitude>
<node name="Spartobacteria"><magnitude><val> 9</val></magnitude>
<node name="Chthoniobacterales"><magnitude><val> 9</val></magnitude>
<node name="Chthoniobacteraceae"><magnitude><val> 9</val></magnitude>
</node>
</node>
</node>
</node>
<node name="Opitutae"><magnitude><val> 114</val></magnitude>
<node name="Opitutae"><magnitude><val> 114</val></magnitude>
<node name="Opitutales"><magnitude><val> 110</val></magnitude>
<node name="Opitutaceae"><magnitude><val> 110</val></magnitude>
</node>
</node>
</node>
</node>
</node>
<node name="Chloroflexi"><magnitude><val> 59</val></magnitude>
<node name="Chloroflexi"><magnitude><val> 59</val></magnitude>
<node name="Ktedonobacteria"><magnitude><val> 27</val></magnitude>
</node>
<node name="Anaerolineae"><magnitude><val> 4</val></magnitude>
<node name="Anaerolineales"><magnitude><val> 4</val></magnitude>
<node name="Anaerolineaceae"><magnitude><val> 4</val></magnitude>

```
</node>
</node>
</node>
<node name="Thermomicrobia"><magnitude><val> 4</val></magnitude>
</node>
</node>
</node>
<node name="Bacteria_I.S."><magnitude><val> 30</val></magnitude>
<node name="Bacteria_I.S."><magnitude><val> 30</val></magnitude>
</node>
</node>
<node name="Fibrobacteres"><magnitude><val> 12</val></magnitude>
<node name="Fibrobacteres"><magnitude><val> 12</val></magnitude>
<node name="Fibrobacteria"><magnitude><val> 12</val></magnitude>
<node name="Fibrobacterales"><magnitude><val> 12</val></magnitude>
<node name="Fibrobacteraceae"><magnitude><val> 12</val></magnitude>
</node>
</node>
</node>
</node>
</node>
<node name="Elusimicrobia"><magnitude><val> 24</val></magnitude>
<node name="Elusimicrobia"><magnitude><val> 24</val></magnitude>
<node name="Elusimicrobia"><magnitude><val> 24</val></magnitude>
</node>
</node>
</node>
<node name="Planctomycetes"><magnitude><val> 19</val></magnitude>
<node name="Planctomycetes"><magnitude><val> 19</val></magnitude>
<node name="Planctomycetacia"><magnitude><val> 19</val></magnitude>
<node name="Planctomycetales"><magnitude><val> 19</val></magnitude>
<node name="Planctomycetaceae"><magnitude><val> 19</val></magnitude>
</node>
</node>
</node>
</node>
</node>
<node name="Gemmatimonadetes"><magnitude><val> 14</val></magnitude>
<node name="Gemmatimonadetes"><magnitude><val> 14</val></magnitude>
<node name="Gemmatimonadetes"><magnitude><val> 14</val></magnitude>
<node name="Gemmatimonadales"><magnitude><val> 14</val></magnitude>
<node name="Gemmatimonadaceae"><magnitude><val> 14</val></magnitude>
</node>
</node>
</node>
</node>
</node>
<node name="Armatimonadetes"><magnitude><val> 5</val></magnitude>
</node>
<node name="WCHB1-60"><magnitude><val> 4</val></magnitude>
<node name="WCHB1-60"><magnitude><val> 4</val></magnitude>
</node>
</node>
</node>
</node>
    </krona></div>
    </body>
</html>
```

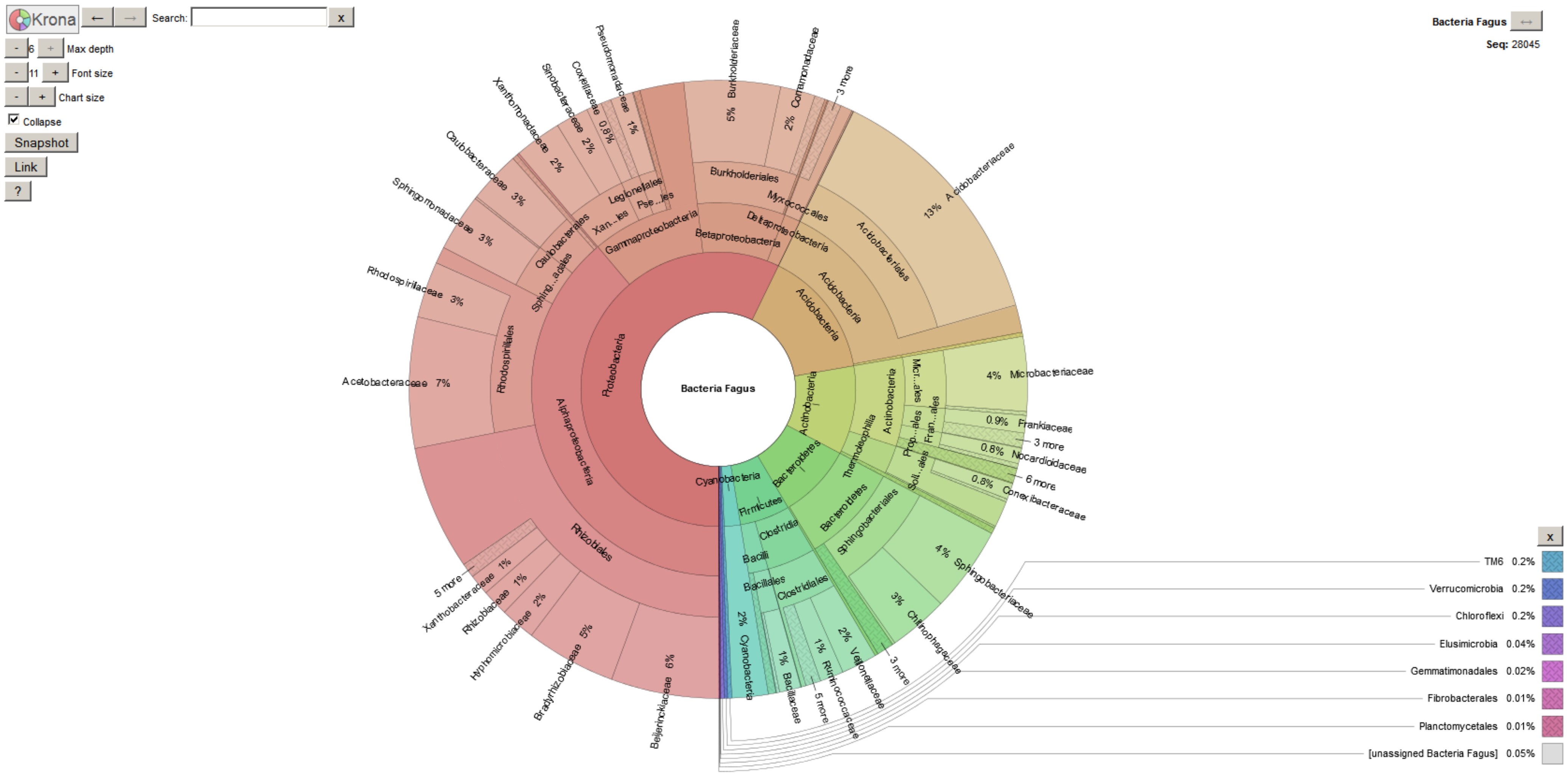

Kronafile SK2: Interactive web-visualization of taxonomic information on *Fagus sylvatica* deadwood logs using Krona (http://sourceforge.net/projects/krona/; Ondov *et al.* 2011 [4]).

Instruction to convert into .html

-> please copy the complete syntax (.html source code) into any text editor and save as "Kronafile SK2.html"
-> the file then opens by double click in your favorite webbrowser

```
<!DOCTYPE html PUBLIC "-//W3C//DTD XHTML 1.0 Strict//EN" "http://www.w3.org/TR/xhtml1/DTD/xhtml1-strict.dtd">
<html xmlns="http://www.w3.org/1999/xhtml" xml:lang="en" lang="en">
    <head>
        <meta charset="utf-8"/>
        <link rel="shortcut icon" href="http://krona.sourceforge.net/img/favicon.ico"/>
        <script id="notfound">window.onload=function(){document.body.innerHTML="Could not get resources from
\"http://krona.sourceforge.net\"."}</script>
        <script src="http://krona.sourceforge.net/src/krona-2.0.js"></script>
    </head>
    <body>
        <img id="hiddenImage" src="http://krona.sourceforge.net/img/hidden.png"  style="display:none"/>
        <noscript>Javascript must be enabled to view this page.</noscript>
        <div style="display:none"><krona>
            <attributes magnitude="magnitude">
                <attribute display="Seq">magnitude</attribute>
            </attributes>
<node name="Bact"><magnitude><val> 28045</val></magnitude>
<node name="Bacteria Fagus"><magnitude><val> 28045</val></magnitude>
<node name="Proteobacteria"><magnitude><val> 16044</val></magnitude>
<node name="Alphaproteobacteria"><magnitude><val> 10891</val></magnitude>
<node name="Alphaproteobacteria"><magnitude><val> 10891</val></magnitude>
<node name="Rhizobiales"><magnitude><val> 6148</val></magnitude>
<node name="Beijerinckiaceae"><magnitude><val> 1573</val></magnitude>
</node>
<node name="Bradyrhizobiaceae"><magnitude><val> 1325</val></magnitude>
</node>
<node name="Hyphomicrobiaceae"><magnitude><val> 514</val></magnitude>
</node>
<node name="Rhizobiaceae"><magnitude><val> 388</val></magnitude>
</node>
<node name="Xanthobacteraceae"><magnitude><val> 303</val></magnitude>
</node>
<node name="Phyllobacteriaceae"><magnitude><val> 95</val></magnitude>
</node>
<node name="Methylocystaceae"><magnitude><val> 68</val></magnitude>
</node>
<node name="Rhodobiaceae"><magnitude><val> 9</val></magnitude>
</node>
<node name="Methylobacteriaceae"><magnitude><val> 42</val></magnitude>
</node>
<node name="Brucellaceae"><magnitude><val> 2</val></magnitude>
</node>
</node>
<node name="Rhodospirillales"><magnitude><val> 2991</val></magnitude>
<node name="Acetobacteraceae"><magnitude><val> 1922</val></magnitude>
</node>
<node name="Rhodospirillaceae"><magnitude><val> 825</val></magnitude>
</node>
</node>
<node name="Sphingomonadales"><magnitude><val> 875</val></magnitude>
<node name="Sphingomonadaceae"><magnitude><val> 842</val></magnitude>
</node>
<node name="Erythrobacteraceae"><magnitude><val> 33</val></magnitude>
</node>
</node>
<node name="Caulobacterales"><magnitude><val> 765</val></magnitude>
<node name="Caulobacteraceae"><magnitude><val> 765</val></magnitude>
</node>
</node>
<node name="Rickettsiales"><magnitude><val> 73</val></magnitude>
<node name="Rickettsiaceae"><magnitude><val> 4</val></magnitude>
</node>
</node>
<node name="Rhodobacterales"><magnitude><val> 6</val></magnitude>
<node name="Rhodobacteraceae"><magnitude><val> 6</val></magnitude>
</node>
</node>
</node>
</node>
<node name="Betaproteobacteria"><magnitude><val> 2129</val></magnitude>
<node name="Betaproteobacteria"><magnitude><val> 2129</val></magnitude>
<node name="Burkholderiales"><magnitude><val> 2074</val></magnitude>
<node name="Burkholderiaceae"><magnitude><val> 1411</val></magnitude>
</node>
<node name="Comamonadaceae"><magnitude><val> 510</val></magnitude>
</node>
<node name="Oxalobacteraceae"><magnitude><val> 113</val></magnitude>
</node>
<node name="Alcaligenaceae"><magnitude><val> 40</val></magnitude>
</node>
</node>
<node name="Nitrosomonadales"><magnitude><val> 32</val></magnitude>
```

```
<node name="Nitrosomonadaceae"><magnitude><val> 31</val></magnitude>
</node>
<node name="Gallionellaceae"><magnitude><val> 1</val></magnitude>
</node>
</node>
<node name="Rhodocyclales"><magnitude><val> 4</val></magnitude>
<node name="Rhodocyclaceae"><magnitude><val> 4</val></magnitude>
</node>
</node>
<node name="Neisseriales"><magnitude><val> 3</val></magnitude>
<node name="Neisseriaceae"><magnitude><val> 3</val></magnitude>
</node>
</node>
</node>
</node>
<node name="Gammaproteobacteria"><magnitude><val> 2629</val></magnitude>
<node name="Gammaproteobacteria"><magnitude><val> 2629</val></magnitude>
<node name="Xanthomonadales"><magnitude><val> 1172</val></magnitude>
<node name="Xanthomonadaceae"><magnitude><val> 690</val></magnitude>
</node>
<node name="Sinobacteraceae"><magnitude><val> 482</val></magnitude>
</node>
</node>
<node name="Pseudomonadales"><magnitude><val> 345</val></magnitude>
<node name="Pseudomonadaceae"><magnitude><val> 332</val></magnitude>
</node>
<node name="Moraxellaceae"><magnitude><val> 13</val></magnitude>
</node>
</node>
<node name="Legionellales"><magnitude><val> 371</val></magnitude>
<node name="Coxiellaceae"><magnitude><val> 225</val></magnitude>
</node>
<node name="Legionellaceae"><magnitude><val> 146</val></magnitude>
</node>
</node>
<node name="Oceanospirillales"><magnitude><val> 64</val></magnitude>
<node name="Halomonadaceae"><magnitude><val> 61</val></magnitude>
</node>
<node name="Oceanospirillaceae"><magnitude><val> 3</val></magnitude>
</node>
</node>
<node name="Enterobacteriales"><magnitude><val> 32</val></magnitude>
<node name="Enterobacteriaceae"><magnitude><val> 32</val></magnitude>
</node>
</node>
</node>
</node>
<node name="Deltaproteobacteria"><magnitude><val> 391</val></magnitude>
<node name="Deltaproteobacteria"><magnitude><val> 391</val></magnitude>
<node name="Myxococcales"><magnitude><val> 368</val></magnitude>
<node name="Polyangiaceae"><magnitude><val> 107</val></magnitude>
</node>
<node name="Haliangiaceae"><magnitude><val> 78</val></magnitude>
</node>
<node name="Cystobacteraceae"><magnitude><val> 11</val></magnitude>
</node>
</node>
<node name="Desulfuromonadales"><magnitude><val> 23</val></magnitude>
</node>
</node>
</node>
</node>
<node name="Acidobacteria"><magnitude><val> 4219</val></magnitude>
<node name="Acidobacteria"><magnitude><val> 4159</val></magnitude>
<node name="Acidobacteria"><magnitude><val> 4159</val></magnitude>
<node name="Acidobacteriales"><magnitude><val> 3756</val></magnitude>
<node name="Acidobacteriaceae"><magnitude><val> 3756</val></magnitude>
</node>
</node>
</node>
</node>
<node name="Holophagae"><magnitude><val> 60</val></magnitude>
<node name="Holophagae"><magnitude><val> 60</val></magnitude>
<node name="Holophagales"><magnitude><val> 41</val></magnitude>
<node name="Holophagaceae"><magnitude><val> 41</val></magnitude>
</node>
</node>
</node>
</node>
</node>
<node name="Bacteroidetes"><magnitude><val> 2467</val></magnitude>
<node name="Bacteroidetes"><magnitude><val> 2421</val></magnitude>
<node name="Sphingobacteriia"><magnitude><val> 2169</val></magnitude>
<node name="Sphingobacteriales"><magnitude><val> 2169</val></magnitude>
<node name="Sphingobacteriaceae"><magnitude><val> 1256</val></magnitude>
</node>
<node name="Chitinophagaceae"><magnitude><val> 873</val></magnitude>
</node>
</node>
```

</node>
<node name="Flavobacteria"><magnitude><val> 82</val></magnitude>
<node name="Flavobacteriales"><magnitude><val> 82</val></magnitude>
<node name="Flavobacteriaceae"><magnitude><val> 82</val></magnitude>
</node>
</node>
</node>
<node name="Bacteroidia"><magnitude><val> 83</val></magnitude>
<node name="Bacteroidales"><magnitude><val> 83</val></magnitude>
<node name="Porphyromonadaceae"><magnitude><val> 51</val></magnitude>
</node>
<node name="Bacteroidaceae"><magnitude><val> 32</val></magnitude>
</node>
</node>
</node>
<node name="Cytophagia"><magnitude><val> 87</val></magnitude>
<node name="Cytophagales"><magnitude><val> 87</val></magnitude>
<node name="Cytophagaceae"><magnitude><val> 85</val></magnitude>
</node>
<node name="Flammeovirgaceae"><magnitude><val> 2</val></magnitude>
</node>
</node>
</node>
</node>
</node>
<node name="Firmicutes"><magnitude><val> 1650</val></magnitude>
<node name="Firmicutes"><magnitude><val> 1650</val></magnitude>
<node name="Clostridia"><magnitude><val> 1168</val></magnitude>
<node name="Clostridiales"><magnitude><val> 1160</val></magnitude>
<node name="Veillonellaceae"><magnitude><val> 524</val></magnitude>
</node>
<node name="Lachnospiraceae"><magnitude><val> 136</val></magnitude>
</node>
<node name="Ruminococcaceae"><magnitude><val> 346</val></magnitude>
</node>
<node name="Clostridiaceae"><magnitude><val> 61</val></magnitude>
</node>
<node name="Peptococcaceae"><magnitude><val> 15</val></magnitude>
</node>
<node name="Christensenellaceae"><magnitude><val> 7</val></magnitude>
</node>
<node name="Eubacteriaceae"><magnitude><val> 4</val></magnitude>
</node>
</node>
<node name="Thermoanaerobacterales"><magnitude><val> 8</val></magnitude>
<node name="Thermoanaerobacteraceae"><magnitude><val> 8</val></magnitude>
</node>
</node>
</node>
<node name="Bacilli"><magnitude><val> 482</val></magnitude>
<node name="Bacillales"><magnitude><val> 369</val></magnitude>
<node name="Bacillaceae"><magnitude><val> 315</val></magnitude>
</node>
<node name="Alicyclobacillaceae"><magnitude><val> 11</val></magnitude>
</node>
<node name="Paenibacillaceae"><magnitude><val> 30</val></magnitude>
</node>
<node name="Sporolactobacillaceae"><magnitude><val> 4</val></magnitude>
</node>
<node name="Staphylococcaceae"><magnitude><val> 2</val></magnitude>
</node>
</node>
<node name="Lactobacillales"><magnitude><val> 113</val></magnitude>
<node name="Streptococcaceae"><magnitude><val> 113</val></magnitude>
</node>
</node>
</node>
</node>
</node>
<node name="Actinobacteria"><magnitude><val> 2939</val></magnitude>
<node name="Actinobacteria"><magnitude><val> 2939</val></magnitude>
<node name="Actinobacteria"><magnitude><val> 2160</val></magnitude>
<node name="Micrococcales"><magnitude><val> 1153</val></magnitude>
<node name="Microbacteriaceae"><magnitude><val> 1096</val></magnitude>
</node>
<node name="Cellulomonadaceae"><magnitude><val> 53</val></magnitude>
</node>
<node name="Intrasporangiaceae"><magnitude><val> 4</val></magnitude>
</node>
</node>
<node name="Frankiales"><magnitude><val> 478</val></magnitude>
<node name="Frankiaceae"><magnitude><val> 257</val></magnitude>
</node>
<node name="Acidothermaceae"><magnitude><val> 131</val></magnitude>
</node>
<node name="Nakamurellaceae"><magnitude><val> 44</val></magnitude>
</node>
<node name="Sporichthyaceae"><magnitude><val> 36</val></magnitude>
</node>

```
</node>
<node name="Propionibacteriales"><magnitude><val> 300</val></magnitude>
<node name="Nocardioidaceae"><magnitude><val> 228</val></magnitude>
</node>
<node name="Propionibacteriaceae"><magnitude><val> 70</val></magnitude>
</node>
</node>
<node name="Corynebacteriales"><magnitude><val> 62</val></magnitude>
<node name="Mycobacteriaceae"><magnitude><val> 50</val></magnitude>
</node>
<node name="Nocardiaceae"><magnitude><val> 12</val></magnitude>
</node>
</node>
<node name="Micromonosporales"><magnitude><val> 78</val></magnitude>
<node name="Micromonosporaceae"><magnitude><val> 78</val></magnitude>
</node>
</node>
<node name="Kineosporiales"><magnitude><val> 38</val></magnitude>
<node name="Kineosporiaceae"><magnitude><val> 38</val></magnitude>
</node>
</node>
<node name="Streptomycetales"><magnitude><val> 18</val></magnitude>
<node name="Streptomycetaceae"><magnitude><val> 18</val></magnitude>
</node>
</node>
<node name="Pseudonocardiales"><magnitude><val> 14</val></magnitude>
<node name="Pseudonocardiaceae"><magnitude><val> 14</val></magnitude>
</node>
</node>
<node name="Streptosporangiales"><magnitude><val> 6</val></magnitude>
<node name="Streptosporangiaceae"><magnitude><val> 6</val></magnitude>
</node>
</node>
</node>
<node name="Thermoleophilia"><magnitude><val> 691</val></magnitude>
<node name="Solirubrobacterales"><magnitude><val> 662</val></magnitude>
<node name="Conexibacteraceae"><magnitude><val> 234</val></magnitude>
</node>
<node name="Solirubrobacteraceae"><magnitude><val> 20</val></magnitude>
</node>
<node name="Patulibacteraceae"><magnitude><val> 25</val></magnitude>
</node>
</node>
<node name="Gaiellales"><magnitude><val> 29</val></magnitude>
</node>
</node>
<node name="Acidimicrobiia"><magnitude><val> 88</val></magnitude>
<node name="Acidimicrobiales"><magnitude><val> 88</val></magnitude>
<node name="Iamiaceae"><magnitude><val> 9</val></magnitude>
</node>
</node>
</node>
</node>
</node>
<node name="Cyanobacteria"><magnitude><val> 530</val></magnitude>
<node name="Cyanobacteria"><magnitude><val> 530</val></magnitude>
</node>
</node>
<node name="Chloroflexi"><magnitude><val> 46</val></magnitude>
<node name="Chloroflexi"><magnitude><val> 46</val></magnitude>
<node name="Ktedonobacteria"><magnitude><val> 25</val></magnitude>
</node>
<node name="Thermomicrobia"><magnitude><val> 4</val></magnitude>
</node>
<node name="Anaerolineae"><magnitude><val> 1</val></magnitude>
<node name="Anaerolineales"><magnitude><val> 1</val></magnitude>
<node name="Anaerolineaceae"><magnitude><val> 1</val></magnitude>
</node>
</node>
</node>
</node>
</node>
<node name="Verrucomicrobia"><magnitude><val> 51</val></magnitude>
<node name="Opitutae"><magnitude><val> 43</val></magnitude>
<node name="Opitutae"><magnitude><val> 43</val></magnitude>
<node name="Opitutales"><magnitude><val> 41</val></magnitude>
<node name="Opitutaceae"><magnitude><val> 41</val></magnitude>
</node>
</node>
</node>
</node>
<node name="Verrucomicrobia"><magnitude><val> 8</val></magnitude>
<node name="Spartobacteria"><magnitude><val> 1</val></magnitude>
<node name="Chthoniobacterales"><magnitude><val> 1</val></magnitude>
<node name="Chthoniobacteraceae"><magnitude><val> 1</val></magnitude>
</node>
</node>
</node>
</node>
```

```
</node>
<node name="TM6"><magnitude><val> 60</val></magnitude>
<node name="TM6"><magnitude><val> 60</val></magnitude>
</node>
</node>
<node name="Elusimicrobia"><magnitude><val> 12</val></magnitude>
<node name="Elusimicrobia"><magnitude><val> 12</val></magnitude>
<node name="Elusimicrobia"><magnitude><val> 12</val></magnitude>
</node>
</node>
</node>
<node name="Fibrobacteres"><magnitude><val> 4</val></magnitude>
<node name="Fibrobacteres"><magnitude><val> 4</val></magnitude>
<node name="Fibrobacteria"><magnitude><val> 4</val></magnitude>
<node name="Fibrobacterales"><magnitude><val> 4</val></magnitude>
<node name="Fibrobacteraceae"><magnitude><val> 4</val></magnitude>
</node>
</node>
</node>
</node>
</node>
<node name="Gemmatimonadetes"><magnitude><val> 5</val></magnitude>
<node name="Gemmatimonadetes"><magnitude><val> 5</val></magnitude>
<node name="Gemmatimonadetes"><magnitude><val> 5</val></magnitude>
<node name="Gemmatimonadales"><magnitude><val> 5</val></magnitude>
<node name="Gemmatimonadaceae"><magnitude><val> 5</val></magnitude>
</node>
</node>
</node>
</node>
</node>
<node name="Planctomycetes"><magnitude><val> 4</val></magnitude>
<node name="Planctomycetes"><magnitude><val> 4</val></magnitude>
<node name="Planctomycetacia"><magnitude><val> 4</val></magnitude>
<node name="Planctomycetales"><magnitude><val> 4</val></magnitude>
<node name="Planctomycetaceae"><magnitude><val> 4</val></magnitude>
</node>
</node>
</node>
</node>
</node>
</node>
</node>
    </krona></div>
    </body>
</html>
```

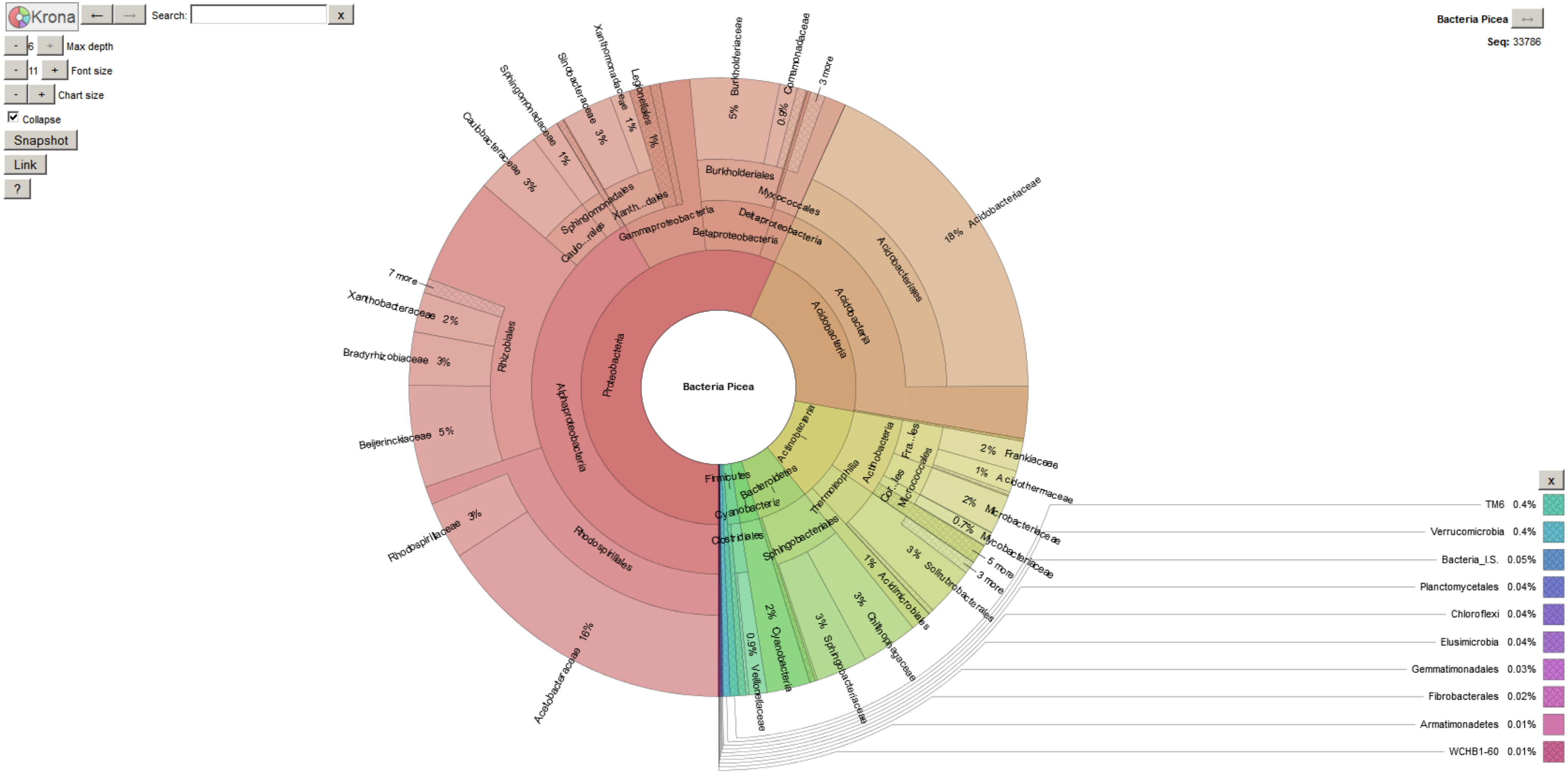

Kronafile SK3: Interactive web-visualization of taxonomic information on *Picea abies* deadwood logs using Krona (http://sourceforge.net/projects/krona/; Ondov *et al.* 2011[4]).

Instruction to convert into .html

-> please copy the complete syntax (.html source code) into any text editor and save as “Kronafile SK3.html”
-> the file then opens by double click in your favorite webbrowser

```
<!DOCTYPE html PUBLIC "-//W3C//DTD XHTML 1.0 Strict//EN" "http://www.w3.org/TR/xhtml1/DTD/xhtml1-strict.dtd">
<html xmlns="http://www.w3.org/1999/xhtml" xml:lang="en" lang="en">
    <head>
        <meta charset="utf-8"/>
        <link rel="shortcut icon" href="http://krona.sourceforge.net/img/favicon.ico"/>
        <script id="notfound">window.onload=function(){document.body.innerHTML="Could not get resources from 
\"http://krona.sourceforge.net\"."}</script>
        <script src="http://krona.sourceforge.net/src/krona-2.0.js"></script>
    </head>
    <body>
        <img id="hiddenImage" src="http://krona.sourceforge.net/img/hidden.png"  style="display:none"/>
        <noscript>Javascript must be enabled to view this page.</noscript>
        <div style="display:none"><krona>
            <attributes magnitude="magnitude">
                <attribute display="Seq">magnitude</attribute>
            </attributes>
<node name="Bact"><magnitude><val> 33786</val></magnitude>
<node name="Bacteria Picea"><magnitude><val> 33786</val></magnitude>
<node name="Proteobacteria"><magnitude><val> 19163</val></magnitude>
<node name="Alphaproteobacteria"><magnitude><val> 14078</val></magnitude>
<node name="Alphaproteobacteria"><magnitude><val> 14078</val></magnitude>
<node name="Rhizobiales"><magnitude><val> 5591</val></magnitude>
<node name="Beijerinckiaceae"><magnitude><val> 1805</val></magnitude>
</node>
<node name="Bradyrhizobiaceae"><magnitude><val> 938</val></magnitude>
</node>
<node name="Xanthobacteraceae"><magnitude><val> 702</val></magnitude>
</node>
<node name="Rhizobiaceae"><magnitude><val> 73</val></magnitude>
</node>
<node name="Hyphomicrobiaceae"><magnitude><val> 122</val></magnitude>
</node>
<node name="Methylobacteriaceae"><magnitude><val> 25</val></magnitude>
</node>
<node name="Phyllobacteriaceae"><magnitude><val> 17</val></magnitude>
</node>
<node name="Rhodobiaceae"><magnitude><val> 4</val></magnitude>
</node>
<node name="Brucellaceae"><magnitude><val> 6</val></magnitude>
</node>
<node name="Methylocystaceae"><magnitude><val> 9</val></magnitude>
</node>
</node>
<node name="Rhodospirillales"><magnitude><val> 6679</val></magnitude>
<node name="Acetobacteraceae"><magnitude><val> 5343</val></magnitude>
</node>
<node name="Rhodospirillaceae"><magnitude><val> 1020</val></magnitude>
</node>
</node>
<node name="Caulobacterales"><magnitude><val> 1179</val></magnitude>
<node name="Caulobacteraceae"><magnitude><val> 1179</val></magnitude>
</node>
</node>
<node name="Sphingomonadales"><magnitude><val> 488</val></magnitude>
<node name="Sphingomonadaceae"><magnitude><val> 487</val></magnitude>
</node>
<node name="Erythrobacteraceae"><magnitude><val> 1</val></magnitude>
</node>
</node>
<node name="Rickettsiales"><magnitude><val> 121</val></magnitude>
<node name="Rickettsiaceae"><magnitude><val> 21</val></magnitude>
</node>
</node>
<node name="Rhodobacterales"><magnitude><val> 3</val></magnitude>
<node name="Rhodobacteraceae"><magnitude><val> 3</val></magnitude>
</node>
</node>
</node>
</node>
<node name="Betaproteobacteria"><magnitude><val> 2133</val></magnitude>
<node name="Betaproteobacteria"><magnitude><val> 2133</val></magnitude>
<node name="Burkholderiales"><magnitude><val> 2051</val></magnitude>
<node name="Burkholderiaceae"><magnitude><val> 1576</val></magnitude>
</node>
<node name="Comamonadaceae"><magnitude><val> 312</val></magnitude>
</node>
<node name="Oxalobacteraceae"><magnitude><val> 145</val></magnitude>
</node>
<node name="Alcaligenaceae"><magnitude><val> 18</val></magnitude>
</node>
</node>
<node name="Nitrosomonadales"><magnitude><val> 22</val></magnitude>
```

```
<node name="Nitrosomonadaceae"><magnitude><val> 16</val></magnitude>
</node>
<node name="Gallionellaceae"><magnitude><val> 6</val></magnitude>
</node>
</node>
<node name="Neisseriales"><magnitude><val> 4</val></magnitude>
<node name="Neisseriaceae"><magnitude><val> 4</val></magnitude>
</node>
</node>
</node>
</node>
<node name="Gammaproteobacteria"><magnitude><val> 2313</val></magnitude>
<node name="Gammaproteobacteria"><magnitude><val> 2313</val></magnitude>
<node name="Xanthomonadales"><magnitude><val> 1242</val></magnitude>
<node name="Sinobacteraceae"><magnitude><val> 892</val></magnitude>
</node>
<node name="Xanthomonadaceae"><magnitude><val> 350</val></magnitude>
</node>
</node>
<node name="Pseudomonadales"><magnitude><val> 119</val></magnitude>
<node name="Pseudomonadaceae"><magnitude><val> 107</val></magnitude>
</node>
<node name="Moraxellaceae"><magnitude><val> 12</val></magnitude>
</node>
</node>
<node name="Legionellales"><magnitude><val> 368</val></magnitude>
<node name="Legionellaceae"><magnitude><val> 175</val></magnitude>
</node>
<node name="Coxiellaceae"><magnitude><val> 193</val></magnitude>
</node>
</node>
<node name="Oceanospirillales"><magnitude><val> 28</val></magnitude>
<node name="Halomonadaceae"><magnitude><val> 27</val></magnitude>
</node>
<node name="Oceanospirillaceae"><magnitude><val> 1</val></magnitude>
</node>
</node>
<node name="Enterobacteriales"><magnitude><val> 27</val></magnitude>
<node name="Enterobacteriaceae"><magnitude><val> 27</val></magnitude>
</node>
</node>
</node>
</node>
<node name="Deltaproteobacteria"><magnitude><val> 639</val></magnitude>
<node name="Deltaproteobacteria"><magnitude><val> 639</val></magnitude>
<node name="Myxococcales"><magnitude><val> 635</val></magnitude>
<node name="Polyangiaceae"><magnitude><val> 150</val></magnitude>
</node>
<node name="Haliangiaceae"><magnitude><val> 85</val></magnitude>
</node>
<node name="Cystobacteraceae"><magnitude><val> 26</val></magnitude>
</node>
</node>
</node>
</node>
</node>
<node name="Acidobacteria"><magnitude><val> 7127</val></magnitude>
<node name="Acidobacteria"><magnitude><val> 7076</val></magnitude>
<node name="Acidobacteria"><magnitude><val> 7076</val></magnitude>
<node name="Acidobacteriales"><magnitude><val> 6155</val></magnitude>
<node name="Acidobacteriaceae"><magnitude><val> 6155</val></magnitude>
</node>
</node>
</node>
</node>
<node name="Holophagae"><magnitude><val> 51</val></magnitude>
<node name="Holophagae"><magnitude><val> 51</val></magnitude>
</node>
</node>
</node>
<node name="Bacteroidetes"><magnitude><val> 2038</val></magnitude>
<node name="Bacteroidetes"><magnitude><val> 2038</val></magnitude>
<node name="Sphingobacteriia"><magnitude><val> 1937</val></magnitude>
<node name="Sphingobacteriales"><magnitude><val> 1937</val></magnitude>
<node name="Chitinophagaceae"><magnitude><val> 995</val></magnitude>
</node>
<node name="Sphingobacteriaceae"><magnitude><val> 905</val></magnitude>
</node>
</node>
</node>
<node name="Flavobacteria"><magnitude><val> 48</val></magnitude>
<node name="Flavobacteriales"><magnitude><val> 48</val></magnitude>
<node name="Flavobacteriaceae"><magnitude><val> 48</val></magnitude>
</node>
</node>
</node>
<node name="Cytophagia"><magnitude><val> 53</val></magnitude>
<node name="Cytophagales"><magnitude><val> 53</val></magnitude>
<node name="Cytophagaceae"><magnitude><val> 37</val></magnitude>
```

</node>
<node name="Flammeovirgaceae"><magnitude><val> 16</val></magnitude>
</node>
</node>
</node>
</node>
</node>
<node name="Actinobacteria"><magnitude><val> 3845</val></magnitude>
<node name="Actinobacteria"><magnitude><val> 3845</val></magnitude>
<node name="Acidimicrobiia"><magnitude><val> 374</val></magnitude>
<node name="Acidimicrobiales"><magnitude><val> 374</val></magnitude>
<node name="Iamiaceae"><magnitude><val> 2</val></magnitude>
</node>
</node>
</node>
<node name="Thermoleophilia"><magnitude><val> 1181</val></magnitude>
<node name="Solirubrobacterales"><magnitude><val> 1093</val></magnitude>
<node name="Conexibacteraceae"><magnitude><val> 197</val></magnitude>
</node>
<node name="Patulibacteraceae"><magnitude><val> 17</val></magnitude>
</node>
<node name="Solirubrobacteraceae"><magnitude><val> 4</val></magnitude>
</node>
</node>
<node name="Gaiellales"><magnitude><val> 88</val></magnitude>
</node>
</node>
<node name="Actinobacteria"><magnitude><val> 2290</val></magnitude>
<node name="Micrococcales"><magnitude><val> 702</val></magnitude>
<node name="Microbacteriaceae"><magnitude><val> 691</val></magnitude>
</node>
<node name="Cellulomonadaceae"><magnitude><val> 9</val></magnitude>
</node>
<node name="Intrasporangiaceae"><magnitude><val> 2</val></magnitude>
</node>
</node>
<node name="Frankiales"><magnitude><val> 989</val></magnitude>
<node name="Acidothermaceae"><magnitude><val> 382</val></magnitude>
</node>
<node name="Frankiaceae"><magnitude><val> 528</val></magnitude>
</node>
<node name="Sporichthyaceae"><magnitude><val> 31</val></magnitude>
</node>
<node name="Nakamurellaceae"><magnitude><val> 25</val></magnitude>
</node>
</node>
<node name="Corynebacteriales"><magnitude><val> 260</val></magnitude>
<node name="Mycobacteriaceae"><magnitude><val> 245</val></magnitude>
</node>
<node name="Nocardiaceae"><magnitude><val> 15</val></magnitude>
</node>
</node>
<node name="Streptomycetales"><magnitude><val> 94</val></magnitude>
<node name="Streptomycetaceae"><magnitude><val> 94</val></magnitude>
</node>
</node>
<node name="Propionibacteriales"><magnitude><val> 142</val></magnitude>
<node name="Nocardioidaceae"><magnitude><val> 124</val></magnitude>
</node>
<node name="Propionibacteriaceae"><magnitude><val> 16</val></magnitude>
</node>
</node>
<node name="Kineosporiales"><magnitude><val> 40</val></magnitude>
<node name="Kineosporiaceae"><magnitude><val> 40</val></magnitude>
</node>
</node>
<node name="Micromonosporales"><magnitude><val> 50</val></magnitude>
<node name="Micromonosporaceae"><magnitude><val> 50</val></magnitude>
</node>
</node>
<node name="Pseudonocardiales"><magnitude><val> 9</val></magnitude>
<node name="Pseudonocardiaceae"><magnitude><val> 9</val></magnitude>
</node>
</node>
</node>
</node>
</node>
<node name="Cyanobacteria"><magnitude><val> 764</val></magnitude>
<node name="Cyanobacteria"><magnitude><val> 764</val></magnitude>
</node>
</node>
<node name="Firmicutes"><magnitude><val> 500</val></magnitude>
<node name="Firmicutes"><magnitude><val> 500</val></magnitude>
<node name="Clostridia"><magnitude><val> 370</val></magnitude>
<node name="Clostridiales"><magnitude><val> 370</val></magnitude>
<node name="Veillonellaceae"><magnitude><val> 315</val></magnitude>
</node>
<node name="Lachnospiraceae"><magnitude><val> 18</val></magnitude>
</node>

<node name="Ruminococcaceae"><magnitude><val> 27</val></magnitude>
</node>
<node name="Clostridiaceae"><magnitude><val> 6</val></magnitude>
</node>
<node name="Peptococcaceae"><magnitude><val> 2</val></magnitude>
</node>
<node name="Christensenellaceae"><magnitude><val> 1</val></magnitude>
</node>
<node name="Eubacteriaceae"><magnitude><val> 1</val></magnitude>
</node>
</node>
</node>
<node name="Bacilli"><magnitude><val> 130</val></magnitude>
<node name="Bacillales"><magnitude><val> 126</val></magnitude>
<node name="Bacillaceae"><magnitude><val> 101</val></magnitude>
</node>
<node name="Paenibacillaceae"><magnitude><val> 20</val></magnitude>
</node>
<node name="Staphylococcaceae"><magnitude><val> 4</val></magnitude>
</node>
<node name="Alicyclobacillaceae"><magnitude><val> 1</val></magnitude>
</node>
</node>
<node name="Lactobacillales"><magnitude><val> 4</val></magnitude>
</node>
</node>
</node>
</node>
<node name="TM6"><magnitude><val> 148</val></magnitude>
<node name="TM6"><magnitude><val> 148</val></magnitude>
</node>
</node>
<node name="Verrucomicrobia"><magnitude><val> 119</val></magnitude>
<node name="Verrucomicrobia"><magnitude><val> 48</val></magnitude>
<node name="Spartobacteria"><magnitude><val> 8</val></magnitude>
<node name="Chthoniobacterales"><magnitude><val> 8</val></magnitude>
<node name="Chthoniobacteraceae"><magnitude><val> 8</val></magnitude>
</node>
</node>
</node>
</node>
<node name="Opitutae"><magnitude><val> 71</val></magnitude>
<node name="Opitutae"><magnitude><val> 71</val></magnitude>
<node name="Opitutales"><magnitude><val> 69</val></magnitude>
<node name="Opitutaceae"><magnitude><val> 69</val></magnitude>
</node>
</node>
</node>
</node>
</node>
<node name="Bacteria_I.S."><magnitude><val> 16</val></magnitude>
<node name="Bacteria_I.S."><magnitude><val> 16</val></magnitude>
</node>
</node>
<node name="Fibrobacteres"><magnitude><val> 8</val></magnitude>
<node name="Fibrobacteres"><magnitude><val> 8</val></magnitude>
<node name="Fibrobacteria"><magnitude><val> 8</val></magnitude>
<node name="Fibrobacterales"><magnitude><val> 8</val></magnitude>
<node name="Fibrobacteraceae"><magnitude><val> 8</val></magnitude>
</node>
</node>
</node>
</node>
</node>
<node name="Planctomycetes"><magnitude><val> 15</val></magnitude>
<node name="Planctomycetes"><magnitude><val> 15</val></magnitude>
<node name="Planctomycetacia"><magnitude><val> 15</val></magnitude>
<node name="Planctomycetales"><magnitude><val> 15</val></magnitude>
<node name="Planctomycetaceae"><magnitude><val> 15</val></magnitude>
</node>
</node>
</node>
</node>
</node>
<node name="Chloroflexi"><magnitude><val> 13</val></magnitude>
<node name="Chloroflexi"><magnitude><val> 13</val></magnitude>
<node name="Anaerolineae"><magnitude><val> 3</val></magnitude>
<node name="Anaerolineales"><magnitude><val> 3</val></magnitude>
<node name="Anaerolineaceae"><magnitude><val> 3</val></magnitude>
</node>
</node>
</node>
<node name="Ktedonobacteria"><magnitude><val> 2</val></magnitude>
</node>
</node>
</node>
<node name="Elusimicrobia"><magnitude><val> 12</val></magnitude>
<node name="Elusimicrobia"><magnitude><val> 12</val></magnitude>
<node name="Elusimicrobia"><magnitude><val> 12</val></magnitude>

```
&lt;/node&gt;
&lt;/node&gt;
&lt;/node&gt;
&lt;node name="Armatimonadetes"&gt;&lt;magnitude&gt;&lt;val&gt; 5&lt;/val&gt;&lt;/magnitude&gt;
&lt;/node&gt;
&lt;node name="Gemmatimonadetes"&gt;&lt;magnitude&gt;&lt;val&gt; 9&lt;/val&gt;&lt;/magnitude&gt;
&lt;node name="Gemmatimonadetes"&gt;&lt;magnitude&gt;&lt;val&gt; 9&lt;/val&gt;&lt;/magnitude&gt;
&lt;node name="Gemmatimonadetes"&gt;&lt;magnitude&gt;&lt;val&gt; 9&lt;/val&gt;&lt;/magnitude&gt;
&lt;node name="Gemmatimonadales"&gt;&lt;magnitude&gt;&lt;val&gt; 9&lt;/val&gt;&lt;/magnitude&gt;
&lt;node name="Gemmatimonadaceae"&gt;&lt;magnitude&gt;&lt;val&gt; 9&lt;/val&gt;&lt;/magnitude&gt;
&lt;/node&gt;
&lt;/node&gt;
&lt;/node&gt;
&lt;/node&gt;
&lt;/node&gt;
&lt;node name="WCHB1-60"&gt;&lt;magnitude&gt;&lt;val&gt; 4&lt;/val&gt;&lt;/magnitude&gt;
&lt;node name="WCHB1-60"&gt;&lt;magnitude&gt;&lt;val&gt; 4&lt;/val&gt;&lt;/magnitude&gt;
&lt;/node&gt;
&lt;/node&gt;
&lt;/node&gt;
&lt;/node&gt;
    &lt;/krona&gt;</div>
    &lt;/body&gt;
&lt;/html&gt;
```

Supplementary References

1. Purahong, W. *et al.* Changes within a single land-use category alter microbial diversity and community structure: Molecular evidence from wood-inhabiting fungi in forest ecosystems. *J. Environ. Manage.* **139**, 109–119 (2014).
2. Hoppe, B. *et al.* Network analysis reveals ecological links between N-fixing bacteria and wood-decaying fungi. *PLoS ONE* **e88141**. doi:10.1371/journal.pone.0088141 (2014).
3. Kahl, T., Mund, M., Bauhus, J. & Schulze, E.D. Dissolved organic carbon from European beech logs: Patterns of input to and retention by surface soil. *Ecoscience* **19**, 1-10 (2012).
4. Ondov, B., Bergmann, N. & Philippy, N. Interactive metagenomic visualization in a Web browser. *BMC Bioinformatics* **12**:385 doi:10.1186/1471-2105-12-385 (2011).
